# Phaeosphaeridiols A–C: Three New Compounds from Undescribed *Phaeosphaeriaceae* sp. SGSF723

**DOI:** 10.3390/jof8111190

**Published:** 2022-11-11

**Authors:** Lu Si, Yao Liu, Tingting Du, Wei Meng, Lijian Xu

**Affiliations:** 1 College of Advanced Agriculture and Ecological Environment, Heilongjiang University, Harbin 150080, China; 2 College of Life Science, Northeast Forestry University, Harbin 150040, China

**Keywords:** antimicrobial compounds, forest litter, Pleosporales, phylogeny

## Abstract

Fungi in forest litter are diverse as decomposers but natural products from these fungi are rarely investigated, especially for their antimicrobial activities against crop diseases. In this study, fungal isolate SGSF723 with antimicrobial activities was cultured. A multi-gene phylogenetic analysis showed SGSF723 was an undescribed species in the family Phaeosphaeriaceae. By bio-guided assay, three new compounds (Phaeosphaeridiols A–C) and two known compounds were purified from the ethyl acetate extract. The structures of Phaeosphaeridiols A–C were elucidated as 2-(2′-butenyl)-5 (3″-pentene)-1,3-benzenediol (**1**), 2-(2′-butenyl)-5-(3″S,4″S-pentane diol)-1,3-benzenediol (**2**), and 3-(4′-(2″-butenyl)-3′,5′-benzenediol phenol)-2-acrylic acid (**3**) by 2D NMR, HRESIMS, and Mosher’s method. Phaeosphaeridiols A–C exhibited moderate or weak antimicrobial activities against plant pathogens by 96-well plate and spore germination assays.

## 1. Introduction

Fungi as natural resources can be applied in agriculture to protect crops [1]. Fungi species are abundant, and over 144,000 fungal species have been described [2,3]; but lots of fungi have never been cultured, and their secondary metabolites also deserve to be investigated extensively, especially newly discovered fungal species.

Forest litters naturally include fallen leaves, dead insects, feces, bacteria, and fungi [4,5]. Some fungi in forest litter play an important role as decomposers in forest ecosystems. Our previous studies showed that forest litter is a promising substrate for the discovery of undescribed and uncultured fungi in general, as exemplified by the recently described *Myxotrichum albicans* [4] and *Parametrhizium* spp. [5].

Phaeosphaeriaceae is a large family in the order Pleosporales and includes plant pathogens, endophytes, and saprobes on plant hosts [6]. Phaeosphaeriaceae was first introduced by Barr in 1979 [7] and subsequently revised by taxonomists [6,8,9,10,11] on the basis of morphological and phylogenetic evidences. The morphological evidences are mainly based on their ascomata (globose or conical, short papillate, small to medium), bitunicate asci, yellowish or brown ascospores, and pycnidial conidiomata. More information on the taxonomy of Phaeospaeriaceae was introduced by Barr in 1979 [7] and Phookamsak et al. in 2014 [6]. The fungi in Phaeosphaeriaceae typically live on herbaceous stems or monocotyledonous leaves, culms, or flowers, but also on woody substrates [7,8,9,10,11]. Currently, it accommodates 84 genera and more than 300 species [3]. Over 100 compounds from over 10 different genera in Phaeosphaeriaceae have been purified and identified (for more information, see Supplementary Table S1). There were 71 compounds with antibacterial, antifungal, or cytotoxic activities, including alkaloids, isocoumarins, quinones, diterpenes, and cyclic peptidesin the genus Phaeosphaeria (Phaeosphaeriaceae) alone [12]. In addition, compounds produced by some genera in Phaeosphaeriaceae, including *Ampelomyces* [13,14], *Edenia* [15,16], *Ophiobolus* [17,18,19,20,21], and *Setophoma* [22,23], were summarized in Supplementary Table S1. In this study, we focus on an undescribed species in Phaeosphaeriaceae.

Plant pathogenic bacteria and fungi cause serious crop yield losses in agriculture globally, such as *Xanthomonas* spp., *Ralstonia solanacearum*, *Alternaria* spp., and *Rhizoctonia solani.* Natural products from microorganisms can be used to prevent and control crop diseases [24,25]. For example, natural products from *Streptomyces* spp., validamycins (jinggangmycins) [26] with antifungal activities, kasugamycin (chunleimycin) with antimicrobial activities [27], and streptomycin with antibacterial activities are used as pesticides [28], and strobilurins [29] originally from fungi are used as fungicides in agriculture. This study aimed to discover novel compounds from an undescribed fungus and investigate the antimicrobial activities of the compounds against plant pathogens.

In this study, we purified and identified three new benzenediols, Phaeosphaeridiols A–C, and two known compounds from an undescribed *Phaeosphaeriaceae* sp. originally derived from forest litter in Greater Hinggan Mountains, China. These new compounds from this undescribed fungal strain exhibited moderate or weak antimicrobial agents against plant pathogens.

## 2. Materials and Methods

### 2.1. General Experimental Procedures

High-performance liquid chromatography (HPLC) was performed on an LC-2A liquid chromatography system (Shimadzu, Shanghai, China). Sephadex LH-20 (GE Healthcare, Stockholm, Sweden) and silica gel (200–300 mesh, Qingdao Haiyang Chemical Co., Ltd., Qingdao, China) were used for column chromatography (CC). Semi-preparative HPLC was performed on a QuikSep high-pressure chromatography system (H&E Co., Ltd., Beijing, China). MeOH and H_2_O used in the HPLC system were chromatographic grade, and all other chemicals were analytical. The NMR spectra were determined on Bruker Avance III 400 instruments (400 MHz for ^1^H and 100 MHz for ^13^C NMR) (Bruker, Fällande, Switzerland). The 1D and 2D NMR spectra were measured on a Bruker 600 spectrometer (600 MHz for ^1^H and 150 MHz for ^13^C). HRESIMS was obtained using a TOF-ESI-MS (Waters Synapt G2, Milford, MA, USA) [30]. (S)- and (R)-phenylglycine methyl ester were bought from Sigma-Aldrich (St. Louis, MO, USA).

### 2.2. Fungal Material

Fungal isolate SGSF723 was isolated from the forest litter of Greater Hinggan Mountains, Heilongjiang province, China. the method of fungal isolation was described by Liang et al. and Gao et al. [4,5]. SGSF723 was deposited in China General Microbiological Culture Collection Center, and the number is CGMCC3.23777. SGSF723 was inoculated to OA (30 g oatmeal, 20 g agar), PDA (200 g potato, 20 g dextrose, 20 g agar), MEA (40 g malt extract, 20 g agar), and YMA (2 g yeast extract, 10 g malt extract 20 g agar) media at different temperatures to observe its morphological characteristics and test whether it can produce spores, respectively. The diameters of the colonies on the different media at different temperatures (15 °C, 20 °C, 25 °C, and 30 °C) for 2 weeks were measured to determine the optimum medium for the colony growth of SGSF723 by a vertical cross-line method. The 6-mm fungal disc from the PDA culture was taken as the inoculum. Three biological repeats were performed for each treatment in the colony growth test. The widths of hyphae were measured under an Olympus BX53 microscope (magnified 400 times) by counting 50 hyphae with different widths.

### 2.3. DNA Extraction, PCR, and Sequencing

DNA extraction of fresh cultures and amplification of the ITS (the nuclear rDNA internal transcribed spacer region containing ITS1-5.8S-ITS4), LSU (the nuclear rDNA large subunit), SSU (the nuclear rDNA small subunit), and TEF (translation elongation factor 1 alpha) loci were performed as described by Liang et al. and Gao et al. [4,5], and DNA sequences were aligned and analyzed by MEGA v. 11.0 [31] and BLAST tool to obtain the close taxa (http://www.ncbi.nlm.nih.gov/, accessed on 1 October 2022).

### 2.4. Phylogenetic Analysis

A multi-gene phylogenetic analysis for SGSF723 and 30 strains of their close species in Phaeosphaeriaceae (Pleosporales) were performed, and *Macroventuria anomochaeta* (Didymellaceae, Pleosporales) was used as an outgroup. The sequences were obtained from GenBank (Table S1) and aligned with MUSCLE [6,32]. A combined dataset of the four loci (ITS, LSU, SSU, and TEF) was produced. A maximum likelihood analysis (ML) of the dataset was performed in RaxmlGUI v2.0 [33,34] using a generalized time reversible (GTR) substitution along with a Gamma distribution, and bootstrap was run in 1000 replicates. Markov chain Monte Carlo (MCMC) has been used to estimate the posterior probability (PP) by MrBayes v. 3.2.4. [35]. Four simultaneous Markov chains were operated for 1,000,000 generations (standard deviation of shared frequencies below 0.01) and the trees were sampled every 1000 generations. The trees were used to determine PP in the majority rule consensus tree [5].

### 2.5. Fermentation, Extraction, and Isolation

The strain SGSF723 was cultured on the PDA for 14 days. Three 6-mm fungal discs from the PDA culture were inoculated to 50-mL potato dextrose broth (PDB) medium and cultured at 180 rpm and 25 °C for 4 days, and then 1 mL of the broth with the strain SGSF723 was transferred to the 500-mL flask with the rice medium (30 g rice and 50 mL H_2_O in each 500-mL flask × 40 flasks, without adjusting pH, and in total, about 4 L rice media for the fermentation) statically culturing at 25 °C for 21 days. After the fermentation, an equal volume of EtOAc (ethyl acetate) was added to extract for 24 h and was then filtrated by a filter paper (NEWSTAR^®^) and concentrated in vacuum to obtain the EtOAc extract (19 g).

The EtOAc extract was first subjected to a silica gel column chromatography (with MeOH/CH_2_Cl_2_ gradient system 0:100, 0.5:100, 1:100, 2:100, 3:100, 5:100, 10:100, 20:100, 50:100, and 100:100) to yield 10 fractions, Fr.s A–J. Then, the fractions were tested for their antibacterial activity against *Ralstonia solanacearum*. Fr.s A, B, G, and H with the antibacterial activity were purified by Sephadex LH20 column chromatography (CH_3_Cl-MeOH) and semi-preparative HPLC (2.5 mL/min, detector UV 210 nm and 254 nm, MeOH-H_2_O) to afford **1** (20 mg, t_R_ = 30 min, from Fr.A), **2** (20 mg, t_R_ = 23 min, from Fr.G), **3** (8 mg, t_R_ = 26 min, from Fr.H), **4** (3 mg, t_R_ = 23 min, from Fr.B), and **5** (3 mg, t_R_ = 23 min, from Fr.A).

### 2.6. Spectroscopic Data

Compound **1**: white solid (MeOH); positive ion; ^1^H NMR (500 MHz, DMSO) and ^13^C NMR (125 MHz, DMSO) data, see Table 1; HRESIMS: *m/z* 233.1526 [M+H]^+^, (calcd for C_15_H_21_O_2_^+^, 233.1536).

Compound **2**: white solid (MeOH); positive ion; ^1^H NMR (500 MHz, DMSO) and ^13^C NMR (125 MHz, DMSO) data, see Table 1; HRESIMS: *m*/*z* 267.1595 [M+H]^+^, (calcd for C_15_H_23_O_4_^+^, 267.1591).

Compound **3**: white solid (MeOH); positive ion; ^1^H NMR (500 MHz, DMSO) and ^13^C NMR (125 MHz, DMSO) data, see Table 1; HRESIMS: *m/z* 235.0956 [M+H]^+^, (calcd for C13H15O4^+^, 235.0964).

### 2.7. Preparation of (R)-MTPA and (S)-MTPA Esters of Compound ***2***

To determine the absolute configuration of compound **2**, (*S*)-MTPA-Cl (5 µL) was reacted with compound **2** (1 mg) at room temperature for 24 h to obtain (*R*)-MTPA ester of compound **2**. (*S*)-MTPA ester of compound **2** was obtained in the same way by using (*R*)-MTPA-Cl. ^1^H NMR data, see Table 2.

### 2.8. MIC Values of Monomeric Compounds

The Minimum Inhibitory Concentrations (MIC) of compounds against *Pseudomonas syringae*, *R. solanacearum*, *Xanthomonas oryzae*, and *X. campestrus* pv. *vesicatoria* were investigated by 96-well plates. The compounds were diluted to different concentrations with a 5% DMSO aqueous solution. The bacterial suspension (2 × 10^5^ CFU/mL) was added to a 96-well plate, and then 100 μL of the compound in the different concentrations was added to each well, respectively. Chlortetracycline was used as a positive control and 5% DMSO as a negative control. The 96-well plate was placed in an incubator at 28 °C and showed results after 24 h. All tests were repeated 3 times.

### 2.9. Inhibition Rate of Spore Germination of Monomeric Compounds

The inhibition rate of spore germination against *Alternaria alternata* was investigated on concave slides. The conidia from *A. alternata* on WA (20 g Agar) medium at 25 °C were scraped off by sterile cotton swabs and diluted with 2% glucose aqueous solutions. The solution of compounds **1**–**5** (final concentration was 125 μg/mL) in 5% DMSO and the spore suspension (1 × 10^6^ spores/mL) were added into the well of the concave slide and incubated for 48 h at 25 °C, respectively. The germination rate of spores was observed under a microscope by counting 100 spores. 5% DMSO was used as a control to calculate the inhibition rate of spore germination. All tests were repeated 3 times.

## 3. Results

### 3.1. Morphological Traits and Phylogenetic Analysis

**Figure 1:**
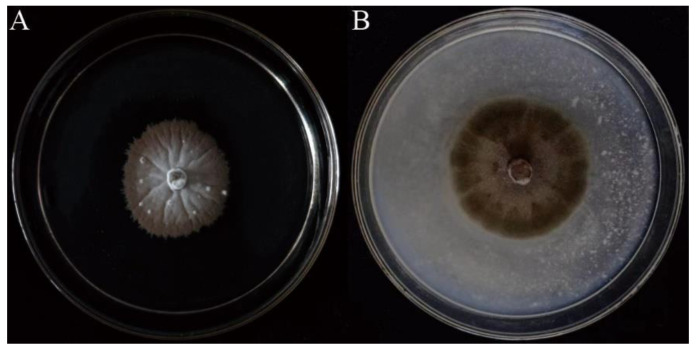
Colonies of strain SGSF723. (**A**) The 5-week-old colony on the PDA. (**B**) The 2-week-old colony on the OA.

To observe the morphological characteristics of strain SGSF723, it was cultured on four different media (MEA, OA, PDA, and YMA) at different temperatures. The optimum condition for the growth was on the OA medium at 25 °C (Supplementary Table S2). There were no conidia or ascospores on any tested media. Its morphological characteristics on the PDA medium were shown in Figure 1. Colonies on PDA white, reaching 14 mm in 2 weeks, filamentous form, velvety, flat elevation, with radial grooves, with brown and erose margin, reverse pale-yellow to brown. Hyphae, hyaline, septate, smooth-walled, 1–4 μm-wide. Conidia or ascospores not observed in any tested cultures.

Since no conidia or ascospores could be observed, the molecular identification was carried out. The DNA sequence similarities on ITS, SSU, LSU, and TEF were investigated as shown in Table 3, respectively. Four different species in Phaeosphaeriaceae were the closest species (Table 3), based on ITS, SSU, LSU, and TEF.

**Figure 2:**
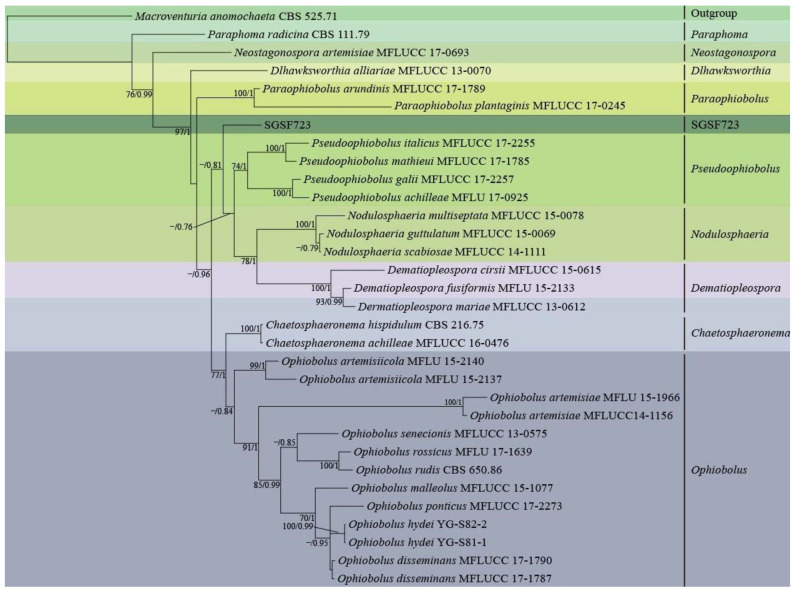
Phylogenetic analysis of strain SGSF723 on ITS, LSU, SSU, and TEF by RAxML and MrBayes; Bootstrap support values ≥ 70% and bayesian posterior probability scores ≥ 0.7 are indicated along branches.

Figure 2 showed phylogenetic analysis on a 2552 bp four-gene dataset (consisting of DNA fragments of 457 bp ITS, 746 bp SSU, 678 bp LSU, and 671 bp TEF) of 30 taxa from seven close genera with SGSF723 in Phaeosphaeriaceae by RAxML using GTR+G model. Five genera in Phaeosphaeriaceae, Pseduoophiobolus, Nodulosphaeria, Dematiopleospora, Ophiobolus, Chaetosphaeronema, and SGSF723 formed a monophyletic clade with the moderate supportive PP value (0.96). Moreover, the relationship between SGSF723 and genera Pseduoophiobolus, Nodulosphaeria, and Dematiopleospora was the closest, and SGSF723 was basal (PP was 0.81) to the clade formed by these three genera. It was difficult to further identify since SGSF723 didn’t produce any spores. SGSF723 could be an undescribed species in Phaeosphaeriaceae, and it was suspected that SGSF723 belonged to a new species or genus in Phaeosphaeriaceae.

### 3.2. Structure Elucidation

In this study, compounds **1**–**5** were isolated from the EtOAc extract of isolate SGSF723 by the bio-guided assay (against *R. solanacearum*). Compounds **1–3** were identified as three new benzenediols, trivially named Phaeosphaeridiol A–C, and two known compounds were identified by HRESIMS and NMR (Supplementary Figures S1–S19). They were 2-(2′-butenyl)-5 (3″-pentene)-1,3-benzenediol (**1**), 2-(2′-butenyl)-5-(3″*S*,4″*S*-pentane diol)-1,3-benzenediol (**2**), and 3-(4′-(2″-butenyl)-3’,5’-benzenediol phenol)-2-acrylic acid (**3**). In addition, the two known compounds **4** and **5** were 4α-methyl-ergosta-6,8(14),22-trien-3β-ol (**4**) and 3-(3-hydroxy-4-(hydroxymethyl) phenyl) acrylic acid (**5**), respectively (Figure 3).

**Figure 3:**
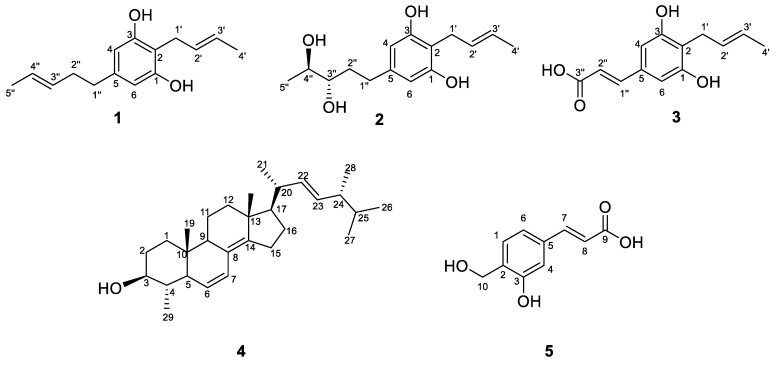
Structures of compounds **1**–**5**.

**Figure 4:**
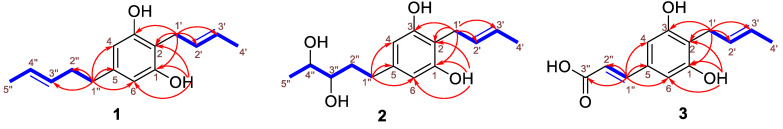
Key HMBC (red arrows) and ^1^H-^1^H COSY (blue-bold) correlations of compounds **1**–**3**.

Compound **1** was obtained as a white solid, and its molecular formula was established as C_15_H_20_O_2_ (six degrees of unsaturation) according to the ion peak cluster at *m/z* 233.1526 [M+H]^+^ in the HRESIMS spectrum. ^1^H and HSQC spectra (Table 1) of compound **1** revealed the presence of two methyls, six olefinic/aromatic methines, three methylenes, as well as two hydroxyls. The carbon spectrum (Table 1) revealed the further presence of four aromatic carbon atom-devoid bound protons. Using the HMBC and COSY data (Figure 4, Figures S3 and S5), the two side chains C–1′ to C–4′ and C–1″ to C–5″ were assembled. Therefore, compound **1** was identified as 2-(2′-butenyl)-5 (3″-pentene)-1,3-benzenediol (Figure 3) and given the trivial name Phaeosphaeridiol A, which is a new compound according to the search within the SciFinder database.

**Figure 5:**
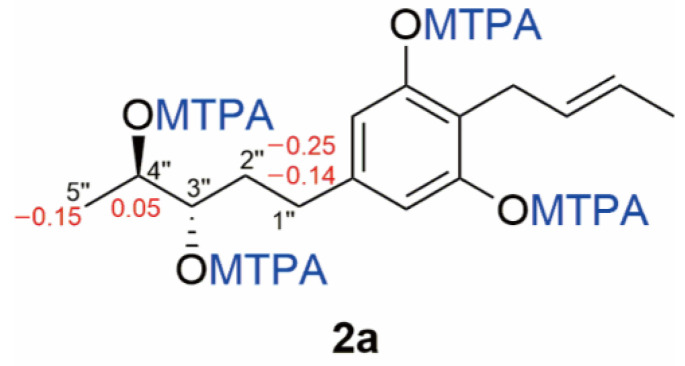
The structures and Δ*δ_S_*-*_R_* values (in ppm) for MTPA esters **2a** of compound **2**.

Compound **2** was obtained as a white solid, and its molecular formula was established as C_15_H_22_O_4_ (five degrees of unsaturation) according to the ion peak cluster at *m/z* 267.1595 [M+H]^+^ in the HRESIMS spectrum. ^1^H and HSQC spectra (Table 1) of compound **2** were similar to compound **1** but with two oxymethines instead of two olefinic methines. The HMBC and COSY data (Figure 4, Figures S8 and S10) revealed 3″-OH and 4″-OH. The relative configuration of compound **2** was elucidated as 2-(2′-butenyl)-5-(3″,4″-pentane diol)-1,3-benzenediol. The absolute configuration of compound **2** was established by Mosher’s method [36]. Esterification of **2** with (*S*)- and (*R*)-MTPA chloride occurred at the hydroxy groups of C–3″ and C–4″ to yield the (*R*)-and (*S*) MTPA esters **2a**, respectively. The observed chemical shift differences Δ*δ* (*δ_S_* − *δ_R_*) (Table 2 and Figure 5) indicated the 3″*S*,4″*S* configuration, and compound **2** was trivially named Phaeosphaeridiol B.

Compound **3** was obtained as a white solid, and its molecular formula was established as C_13_H_14_O_4_ (seven degrees of unsaturation) according to the ion peak cluster at *m/z* 235.0956 [M+H]^+^ in the HRESIMS spectrum. ^1^H, ^13^C and HSQC spectra (Table 1) of compound **3** were similar to compound **1** but one of the side chains (C–1″ to C–5″) in compound **1** was changed to an acrylic acid group with two olefinic methines (C–1′’ and C–2″) and carboxyl (C–3″) in compound **3**. Both the double bond between C–2′ and C–3′ and the double bond between C–1″ and C–2″ are in a trans configuration based on their coupling constants (Table 1). The HMBC and COSY data (Figure 4, Figures S13 and S15) also indicated the structure of compound **3** as shown in Figure 3, and it was given the trivial name Phaeosphaeridiol C, whose systematic IUPAC name is 3-(4′-(2″-butenyl)-3′,5′-benzenediol phenol)-2-acrylic acid.

Compounds **4** and **5** were identified as two known compounds, 4α-methyl-ergosta-6,8(14),22-trien-3β-ol (**4**) and 3-(3-hydroxy-4-(hydroxymethyl) phenyl) acrylic acid (**5**). on the basis of their spectroscopic features (Supplementary Figures S16–S19) and by comparison with the published data in the literature [37,38].

### 3.3. Antimicrobial Activity Assay of Compounds

Table 4 showed that three new compounds and two known compounds had moderate antibacterial activities against four Gram-negative plant-pathogenic bacteria. Compound **1** had stronger antibacterial activities against two species of *Xanthomonas* (MICs, 31.25) than compounds **2** and **3**. In addition, the antifungal activity of compounds **1**–**5** (at 125 µg/mL) against *A. alternata* was also investigated by inhibition of spore germination. The inhibition rates of spore germination of compounds **1**–**5** were 16.87 ± 7.63%, 30.23 ± 4.87%, 35.71 ± 4.65%, 44.92 ± 12.42%, and 30.33 ± 10.95%.

## 4. Discussion

Fungal isolate SGSF723 was isolated and cultured from forest litter. In preliminary antimicrobial screening, the EtOAc extract of SGSF723 showed stronger antifungal and antibacterial activities against plant pathogens than other tested isolates from the forest litter (Supplementary Tables S3 and S4). Therefore, SGSF723 was further investigated. The similarities of sequences ITS, SSU, LSU, and TEF showed SGSF723 was close to four species in four different genera in *Phaeosphaeriacea*. Further, the multi-gene phylogenetic analysis for SGSF723 showed SGSF723 was in the family Phaeosphaeriaceae and is a sister group to the clade formed by *Pseduoophiobolus*, *Nodulosphaeria*, and *Dematiopleospora*. Except for the phylogenetic evidences, genera in Phaeosphaeriacea were divided by their morphological traits on conidia and conidiomata for asexual genera or ascospores and ascomata for sexual genera [6,32]. SGSF723 could be an undescribed species in *Phaeosphaeriaceae* according to our phylogenetic analysis, although there are no conidia or ascospores in any different cultures of SGSF723.

There are 84 genera and over 300 species in Phaeosphaeriaceae. Many natural compounds from the genera in Phaeosphaeriaceae were isolated and identified [19,39]. For example, ophiobolide A–C from *Ophiobolus* exhibited an antifungal activity against *Cochliobolus miyabeanus* [19], and Hispidulones and resorcylic acid lactones from *Chaetosphaeronema hispidulum* exhibited anticancer activities [39]. However, many genera in Phaeosphaeriaceae have never been reported for their natural compounds. According to the search in Dictionary of Natural Products, there are no reports about natural products from the genera *Nodulosphaeria*, *Dematiopleospora*, and *Pseudoophiobolus,* which were close to SGSF723 and formed a sister clade to SGSF723. Three new compounds with antibacterial and antifungal activities, Phaeosphaeridiols A–C, were isolated and identified from an undescribed species in Phaeosphaeriaceae (Pleosporales). The structures of Phaeosphaeridiols A–C contain 1,3-benzendiol with two side chains at C–2 and C–5, and are similar to a known compound, stemphol, which is produced by two fungal species in Pleosporaceae (Pleosporales), *Stemphylium majusculum,* and *Pleospora herbarum* [40]. Stemphol also has antibacterial and antifungal activities against plant pathogens [40]. It implied that Phaeosphaeridiols A–C and Phaeosphaeridiol-like compounds (1,3-benzendiol with two side chains at C–2 and C–5) could have antimicrobial activities against plant pathogens.

In our antimicrobial tests against four plant pathogenic bacteria and one plant pathogenic fungus, only Phaeosphaeridiol A showed moderate antibacterial activities (MIC was 31.25 μg/mL) against two strains in *Xanthomonas* genus (Table 4). The selective antibacterial activities of Phaeosphaeridiol A against *Xanthomonas* spp. probably related to the double bond between C–3″ and C–4″ in comparison with Phaeosphaeridiol B, because this double bond changed to the single bond with two hydroxyl groups in Phaeosphaeridiol B, and the antibacterial activities of Phaeosphaeridiol B against *Xanthomonas* spp. were significantly weaker than for Phaeosphaeridiol A. Because of the moderate or weak antibacterial activities, Phaeosphaeridiols A–C cannot be directly developed as pesticides. However, the structure–activity relationship of more Phaeosphaeridiol-like compounds (1,3-benzendiol with two side chains at C–2 and C–5) could deserve further investigation in the future.

## 5. Conclusions

Three new compounds (Phaeosphaeridiols A–C) and two known compounds were purified from undescribed *Phaeosphaeriaceae* sp. SGSF723, originally derived from forest litter. Phaeosphaeridiol A showed moderate antibacterial activities against plant-pathogenic *Xanthomonas* spp., and the other compounds showed weak antimicrobial activities.

## Figures and Tables

**Table 1:** Spectrum data of three new compounds.

Pos.	1		2		3	
	*δ*_C_ *^b^*	*δ*_H_*^a^*, Mult (*J* in Hz)	*δ*_C_ *^b^*	*δ*_H_*^a^*, Mult (*J* in Hz)	*δ*_C_ *^b^*	*δ*_H_*^a^*, Mult (*J* in Hz)
1	110.8		110.5		116.5	
2	155.7		155.7		156.2	
3	110.8		110.5		116.5	
4	106.2	6.10, s	106.1	6.11, s	106.1	6.51, s
5	139.8		140.9		132.3	
6	106.2	6.10, s	106.1	6.11, s	106.1	6.51, s
1′	25.7	3.11, dt (6.5, 1.5)	25.7	3.10, dt (6.5, 1.5)	25.8	3.17, dt (6.0, 1.5)
2′	129.9	5.47, quintq (15.0, 6.5, 1.5)	130.0	5.45, quintq (15.0, 6.5, 1.5)	129.0	5.46, quintq (15.0, 6.5, 1.5)
3′	123.3	5.32, sextt (15.0, 6.5, 1.5)	123.1	5.31, sextt (15.0, 6.5, 1.5)	124.0	5.35, sextt (15.0, 6.5, 1.5)
4′	17.8	1.55, dq (6.0, 1.5)	17.6	1.55, dq (6.5, 1.5)	17.6	1.56, dq (6.5, 1.5)
1″	35.3	2.38, dd (9.0, 6.5)	31.7	2.54, m 2.30, ddd (13.5, 10.5, 6.5)	144.4	7.32, d (16.0)
2″	33.8	2.16, m	34.8	1.72, dddd (13.5, 10.5, 6.5, 3.0)	117.5	6.15, d (16.0)
3″	130.8	5.43, m	74.2	3.14, dq (9.0, 3.0)	167.5	
4″	124.7	5.43, m	69.8	3.36, m		
5″	17.6	1.60, m	19.4	1.03, d (6.0)		
1-OH		8.86, s		8.82, s		9.32, s
3-OH		8.86, s		8.82, s		9.32, s
3″-OH				4.33, d (6.0)		
4″-OH				4.34, d (6.0)		
3″-COOH						12.29, s

**Table 2:** ^1^H-NMR spectroscopic data of (*S*)- and (*R*)- esters derivatives of compound **2**.

Pos.	*δ_S_*	*δ_R_*	Δ*δ* (*δ_S_* − *δ_R_*)
4	6.73	6.83	−0.10
6	6.73	6.83	−0.10
1′	2.90, 2.82	2.91, 2.85	−0.01, −0.03
2′	5.08	5.09	−0.01
3′	4.91	4.92	−0.01
4′	1.46	1.47	−0.01
1″	2.42	2.67, 2.56	−0.25, −0.14
2″	1.76	1.97, 1.90	−0.21, −0.14
3″	5.27	5.27	0
4″	5.33	5.28	0.05
5″	1.18	1.33	−0.15

**Table 3:** Sequence similarities with their closest-known species of strain SGSF723.

Sequence	Closest Species	Similarity	Coverage	Accession No.*
ITS	*Chaetosphaeronema* *achilleae*	95.30%	97%	ON754203
LSU	*Ophiobolus hydei*	99.38%	100%	MK981304
SSU	*Nodulosphaeria modesta*	100%	98%	KM434294
TEF	*Didymocyrtis cladoniicola*	96.36%	99%	LT797117

Note: * means data from GenBank.

**Table 4:** Minimum inhibitory concentration of compounds (μg/mL).

Compound	*R. solanacearum*	*X. oryzae*	*X. campestrus*	*P. syringae*
compound **1**	250	31.25	31.25	250
compound **2**	125	500	>500	>500
compound **3**	500	250	500	125
compound **4**	>500	250	>500	>500
compound **5**	>500	500	>500	>500
Chlortetracycline	0.98	0.98	1.96	3.92

## Data Availability

Not applicable.

## Supplementary Materials

The following supporting information can be downloaded at: https://www.mdpi.com/article/10.3390/jof8111190/s1, Table S1. Compounds from different genera (Ampelomyces, Edenia, Ophiobolus, and Setophoma) in Phaeosphaeriaceae; Table S2. Diameters (mm) of SGSF723 colonies on different media at different temperatures; Table S3. Antimicrobial activities (diameters of inhibition zones) of crude extracts from different fungal strains; Table S4. ITS sequence similarities with their closest known species of different fungal strains; Figure S1: ^1^H NMR spectrum (500 MHz, DMSO-d) of compound **1**; Figure S2: ^13^C NMR spectrum (125 MHz, DMSO-d) of compound **1**; Figure S3: COSY NMR spectrum (500 MHz, DMSO-d) of compound **1**; Figure S4: HSQC NMR spectrum (500 MHz, DMSO-d) of compound **1**; Figure S5: HMBC NMR spectrum (500 MHz, DMSO-d) of compound **1**; Figure S6: ^1^H NMR spectrum (500 MHz, DMSO-d) of compound **2**; Figure S7: ^13^C NMR spectrum (125 MHz, DMSO-d) of compound **2**; Figure S8: COSY NMR spectrum (500 MHz, DMSO-d) of compound **2**; Figure S9: HSQC NMR spectrum (500 MHz, DMSO-d) of compound **2**; Figure S10: HMBC NMR spectrum (500 MHz, DMSO-d) of compound **2**; Figure S11: ^1^H NMR spectrum (500 MHz, DMSO-d) of compound **3**; Figure S12: ^13^C NMR spectrum (125 MHz, DMSO-d) of compound **3**; Figure S13: COSY NMR spectrum (500 MHz, DMSO-d) of compound **3**; Figure S14: HSQC NMR spectrum (500 MHz, DMSO-d) of compound **3**; Figure S15: HMBC NMR spectrum (500 MHz, DMSO-d) of compound **3**; Figure S16: ^1^H NMR spectrum (400 MHz, Chloroform-d) of compound **4**; Figure S17: ^13^C NMR spectrum (100 MHz, Chloroform-d) of compound **4**; Figure S18: ^1^H NMR spectrum (400 MHz, Methanol-D4) of compound **5**; Figure S19: ^13^C NMR spectrum (100 MHz, Methanol-D4) of compound **5**; Table S5: Accession numbers of sequences used in the phylogenetic analyses.
