# Supplementary material for: Phaeosphaeridiols A–C: Three New Compounds from Undescribed Phaeosphaeriaceae sp. SGSF723
Source: J Fungi (Basel). 2022 Nov 11;8(11):1190. doi: 10.3390/jof8111190 (PMC9697633; doi:10.3390/jof8111190)
Supplement: Supplementary file 1 [file jof-08-01190-s001.zip › jof-1988485-supplementary.pdf]

Supporting Information for

Phaeosphaeridiols A–C: Three New Compounds from Undescribed Phaeosphaeriaceae sp. SGSF723

Lu Si [1,†], Yao Liu [1,†], Tingting Du [1,†], Wei Meng [2], and Lijian Xu [1,*]

[1] College of Advanced Agriculture and Ecological Environment, Heilongjiang University, Harbin 150080, China
[2] College of Life Science, Northeast Forestry University, Harbin 150040, China
* Correspondence: xulijian@hlju.edu.cn or xulijiancau@gmail.com
† These authors contributed equally to this work.

Table S1. Compounds from different genera (*Ampelomyces*, *Edenia*, *Ophiobolus*, and *Setophoma*) in Phaeosphaeriaceae.

Genus	Chemical name	Molecular Formula	References
Ampelomyces	Altersolanol A	$C_{16}H_{16}O_8$	[13]
	8-Hydroxy-3-(2-hydroxypropyl)-6-methoxy-1H-2-benzopyran-1-one; (S)-form, 3'-Hydroxy, O-de-Me	$C_{12}H_{12}O_6$	[13]
	8-Hydroxy-3-(2-hydroxypropyl)-6-methoxy-1H-2-benzopyran-1-one; (S)-form, 3',3'-Dichloro, O-de-Me	$C_{12}H_{10}Cl_2O_5$	[13]
	8-Hydroxy-3-(2-hydroxypropyl)-6-methoxy-1H-2-benzopyran-1-one; (S)-form	$C_{13}H_{14}O_5$	[13]
	4-Hydroxy-6-(2-hydroxypropyl)-3-methyl-2H-pyran-2-one; (ξ)-form, 2'-Ac (Ampelopyrone)	$C_{11}H_{14}O_5$	[13]
	Tetrahydroaltersolanol B; 1β,4β-Dihydroxy	$C_{16}H_{20}O_8$	[13]
	1,2,6,8-Tetrahydroxy-3-methylanthraquinone; 6-Me ether, 2-O-sulfate	$C_{16}H_{12}O_9S$	[13]
	1,3,7-Trihydroxy-6-methylanthraquinone; 3-Me ether, 7-O-sulfate	$C_{16}H_{12}O_8S$	[13]
	2,5-Dihydroxy-3-mercaptobenzyl alcohol; S-Me,S-oxide	$C_8H_{10}O_4S$	[14]
	2,3-Epoxy-5-methyl-1,4-cyclohexanediol; (1R,2R,3S,4R,5R)-form	$C_7H_{12}O_3$	[14]
	4-Hydroxy-6-methyl-2-cyclohexen-1-one; (4R,6S)-form	$C_7H_{10}O_2$	[14]
	5-Methyl-1,2,3,4-cyclohexanetetrol; (1S,2R,3R,4S,5S)-form, 2-O-(2-Hydroxy-6-methylbenzoyl) (Ampelomin G)	$C_{15}H_{20}O_6$	[14]
	5-Methyl-1,2,4-cyclohexanetriol; (1R,2S,4R,5R)-form	$C_7H_{14}O_3$	[14]
	5-Methyl-1,2,4-cyclohexanetriol; (1R,2S,4S,5R)-form	$C_7H_{14}O_3$	[14]
	5-Methyl-1,2,4-cyclohexanetriol; (1S,2R,4R,5R)-form	$C_7H_{14}O_3$	[14]
	5-Methyl-1,2,4-cyclohexanetriol; (1S,2S,4R,5S)-form	$C_7H_{14}O_3$	[14]
Edenia	Palmarumycin CP_1; 2,3-Dihydro	$C_{20}H_{14}O_4$	[15]
	Palmarumycin CP_1; 2,3-Dihydro, 4α-alcohol, 4-Me ether	$C_{21}H_{18}O_4$	[15]
	Preussomerin A; 2,3-Deepoxy, 2',3'-dihydro, 1,1'-diketone	$C_{20}H_{14}O_6$	[15]
	Palmarumycin CP_1; 2,3-Dihydro, 8-hydroxy	$C_{20}H_{14}O_5$	[15]
	Palmarumycin CP_1; 2,3-Dihydro, 8-hydroxy, 4α-alcohol, 4-Me ether (Palmarumycin EG1)	$C_{21}H_{18}O_5$	[16]
	Preussomerin A; 2,3-Deepoxy, 2',3'-dihydro, 3'α-hydroxy, 1,1'-diketone	$C_{20}H_{14}O_7$	[16]

	Preussomerin A; 2,3-Deepoxy, 2',3'-dihydro, 3'α-methoxy, 1,1'-diketone	$C_{21}H_{16}O_7$	[16]
	Preussomerin A; 2,3-Deepoxy, 1,1'-diketone	$C_{20}H_{12}O_6$	[16]
Ophiobolus	Achaetolide	$C_{16}H_{28}O_5$	[17]
	7,18-Ophioboladien-3-ol; 3α-form	$C_{25}H_{42}O$	[18]
	Ophiobolide A	$C_{16}H_{30}O_6$	[19]
	Ophiobolide B	$C_{16}H_{28}O_6$	[19]
	Ophiobolide C	$C_{16}H_{28}O_6$	[19]
	Ophiobolin A	$C_{25}H_{36}O_4$	[20]
	Vermisporin	$C_{25}H_{37}NO_4$	[21]
Setophoma	Blennolide A; 3,4*a*-Diepimer, 1-deoxy	$C_{16}H_{16}O_6$	[22]
	Blennolide D; 3'-Deoxy	$C_{16}H_{16}O_7$	[22]
	Blennolide D; 2-Epimer, 3'-deoxy	$C_{16}H_{16}O_7$	[22]
	Blennolide G; (-)-form	$C_{32}H_{30}O_{14}$	[22]
	Blennolide G; (-)-form, 4'',5''-Diepimer	$C_{32}H_{30}O_{14}$	[22]
	Blennolide G; (-)-form, 4,4'',5''-Triepimer	$C_{32}H_{30}O_{14}$	[22]
	3,4-Dihydro-6,8-dihydroxy-3-propyl-1*H*-2-benzopyran-1-one; (*S*)-form, 1'*S*-Hydroxy	$C_{12}H_{14}O_5$	[22]
	Penicillixanthone A	$C_{32}H_{30}O_{14}$	[22]
	Lecanorin B; 2'-Demethyl	$C_{16}H_{16}O_5$	[23]
	Lecanorin B; 3-Demethyl	$C_{16}H_{16}O_5$	[23]
	Lecanorin B; 3-Demethyl, 4'-methyl	$C_{17}H_{18}O_5$	[23]

Table S2. Diameters (mm) of SGSF723 colonies on different media at different temperatures.

	OA	PDA	YMA	MEA
15 °C	22.00±1.41c	19.00±1.41b	16.33±1.25a	18.00±3.56a
20 °C	27.00±2.83b	23.67±1.70a	19.00±2.16a	21.00±0.82a
25 °C	37.33±0.47a	14.33±0.47c	12.67±0.47b	10.00±1.41b
30 °C	6.33±0.47d	4.00±0.82d	4.67±0.47c	5.00±0.00c

Note: The values mean average ± standard deviation. The letters "a–d" indicate significant differences (at the 0.05 level) in the diameters of colonies on the same media at different temperatures.

Table S3. Antimicrobial activities (diameters of inhibition zones) of crude extracts from different fungal strains.

Strain No.	*R. solanacearum*			*X. oryzae*			*P. syringae*			*A. alternate*		
	**Y**	**R**	**A**	**Y**	**R**	**A**	**Y**	**R**	**A**	**Y**	**R**	**A**
SGSF708	—	—	—	+	+	+	—	—	—	—	+	—
SGSF710	—	—	—	—	+	+	—	—	—	—	—	—
SGSF723	—	+++	++	—	++	—	—	++	+	—	+++	—
SGSF726	+	—	—	—	—	—	—	—	—	+	—	—
SGSF728	—	—	++	—	+	++	—	—	—	+	+	—

Note: "—" means no inhibition effect. "+" means the diameters of inhibition zones are 1–5 mm. "++" means the diameters of inhibition zones are 6–8 mm. "+++" means the diameters of inhibition zones are more than 8 mm. "Y", "R", and "A" represent YES, Rice, and PDA media, respectively.

Table S4. ITS sequence similarities with their closest known species of different fungal strains.

Strain No.	Closest strain*	Similarity*	Coverage	Accession No.*
SGSF708	*Polyphilus sieberi*	94.21%	100%	MG719688
SGSF710	*Coleophoma cylindrospora*	93.91%	97%	MW192862
SGSF723	*Chaetosphaeronema achilleae*	95.30%	97%	ON754203
SGSF726	*Piniphoma wesendahlina*	93.43%	100%	NR_163375
SGSF728	*Nematostoma parasiticum*	95.54%	100%	MT547815

Note: "*" means data from GenBank.

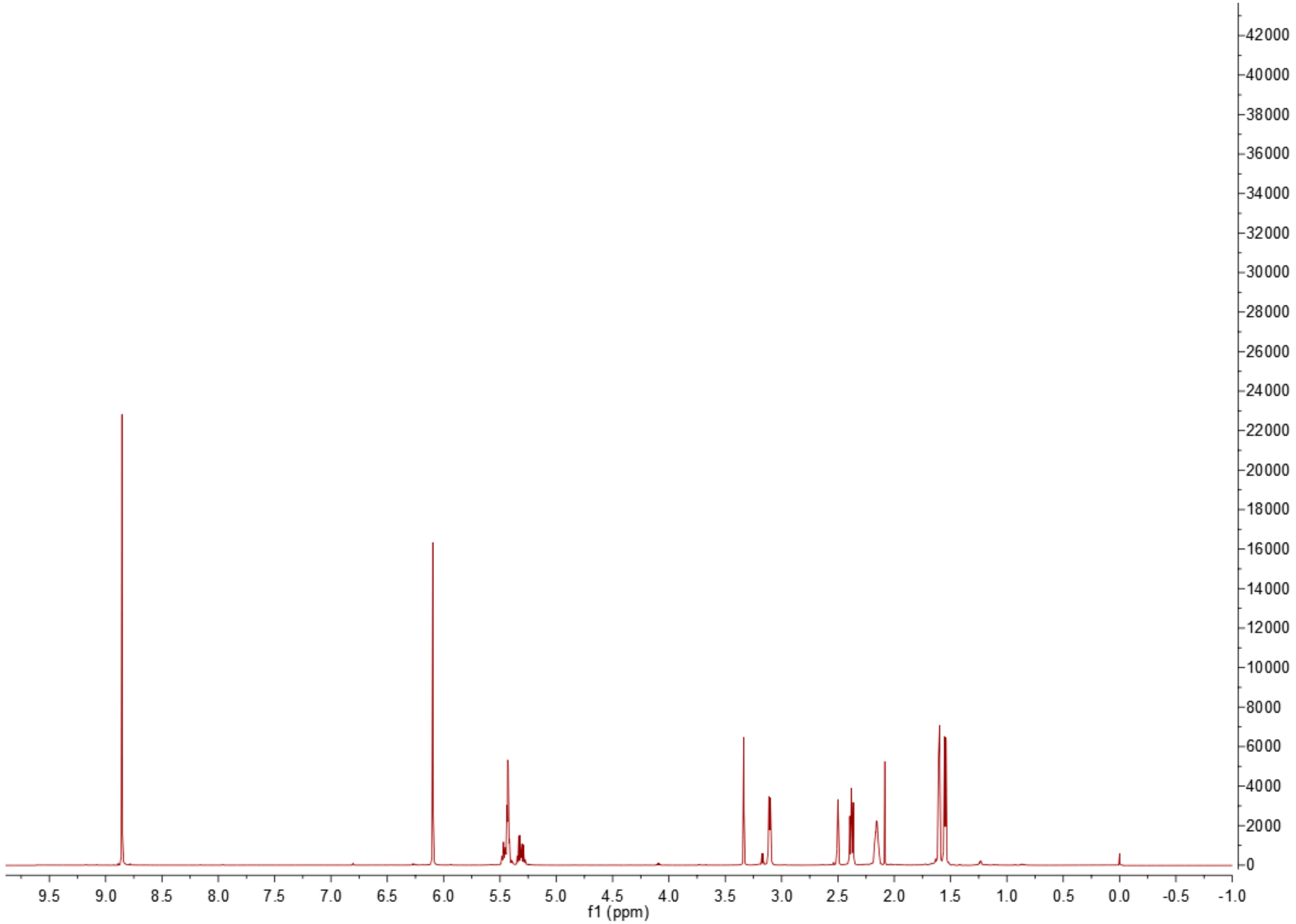

Figure S1: ^{1}H NMR spectrum (500 MHz, DMSO-d) of compound 1.

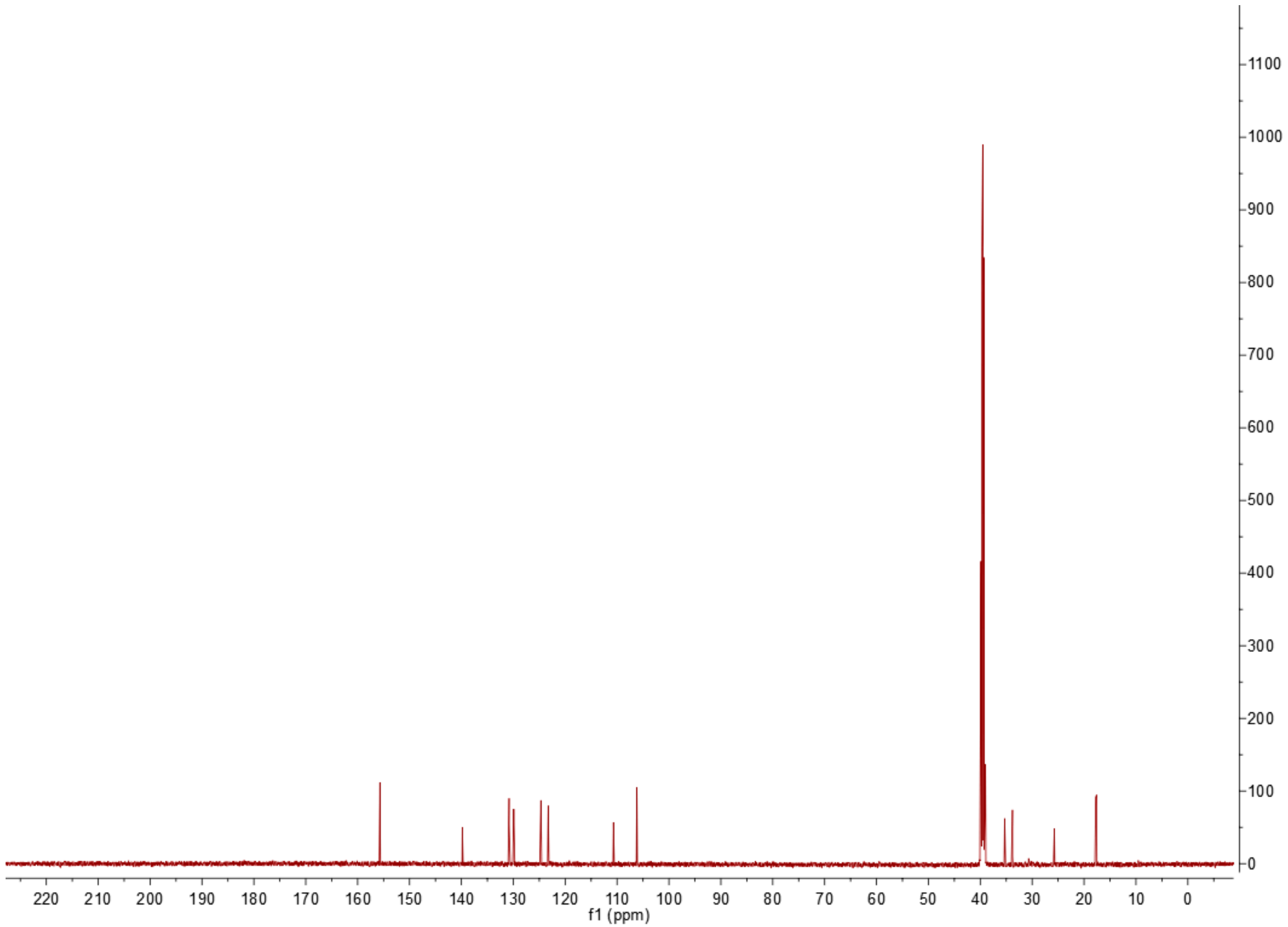

Figure S2: ^{13}C NMR spectrum (125 MHz, DMSO-d) of compound 1.

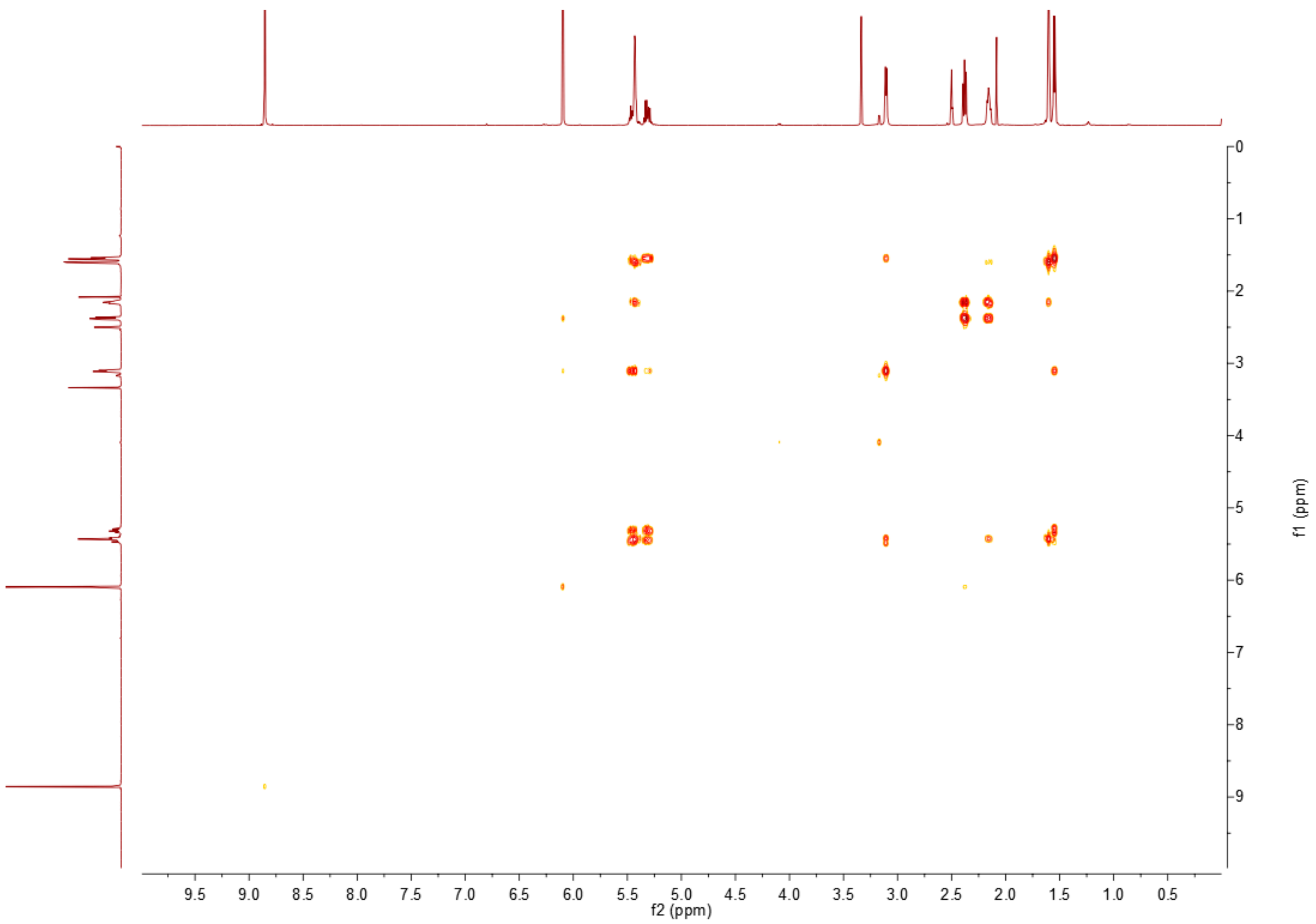

Figure S3: COSY NMR spectrum (500 MHz, DMSO -d) of compound 1.

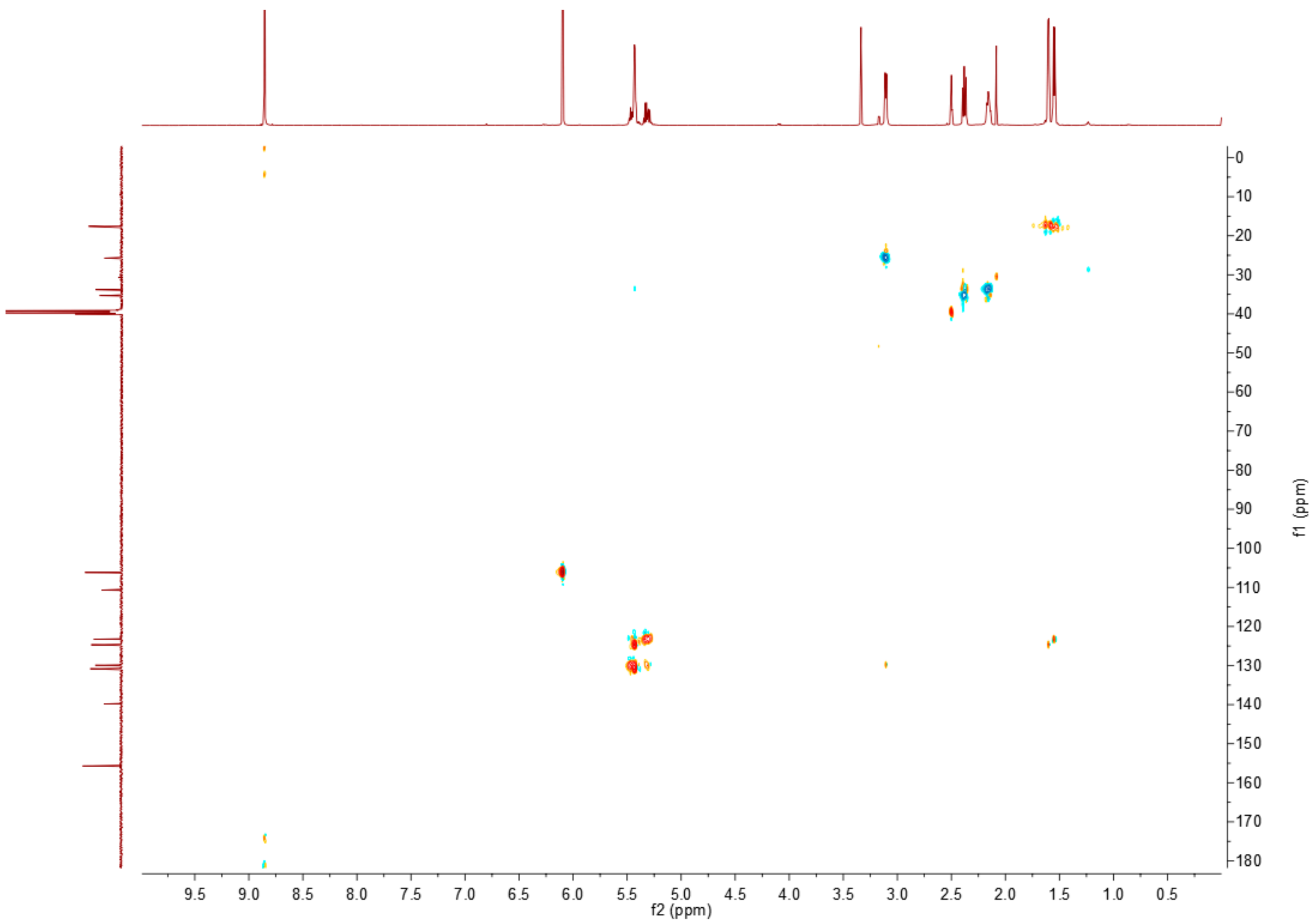

Figure S4: HSQC NMR spectrum (500 MHz, DMSO-d) of compound 1.

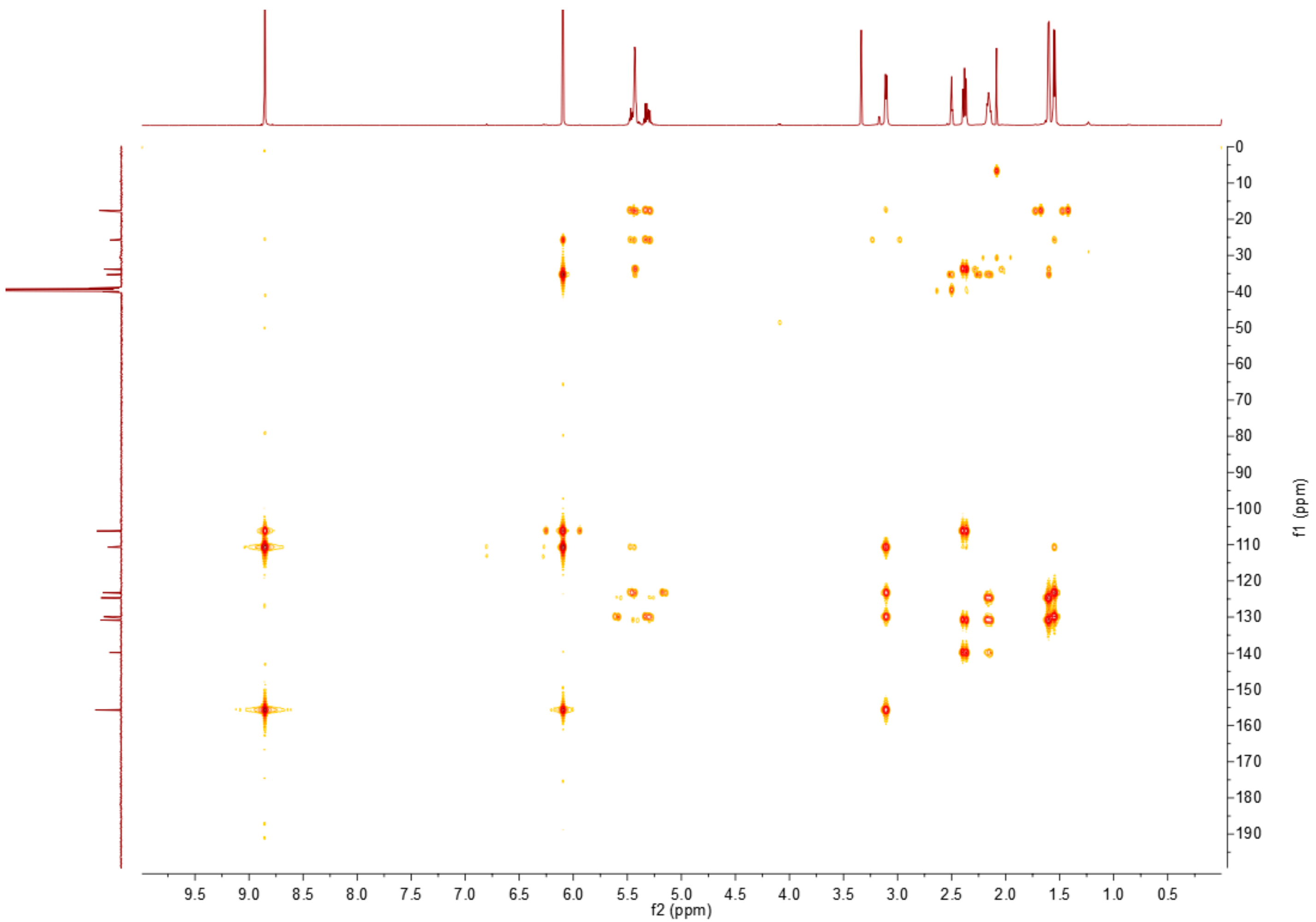

Figure S5: HMBC NMR spectrum (500 MHz, DMSO-d) of compound 1.

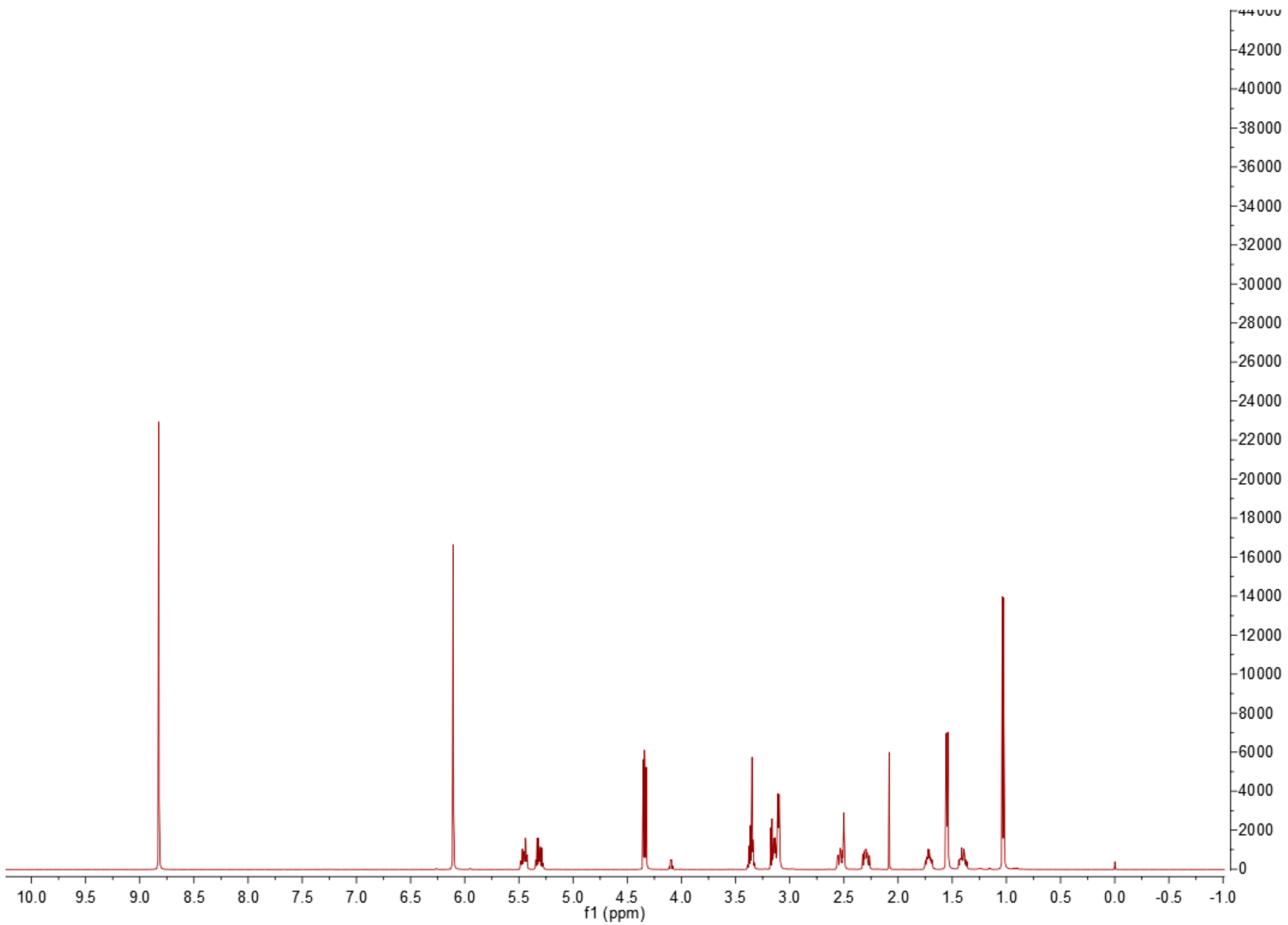

Figure S6: ^{1}H NMR spectrum (500 MHz, DMSO-d) of compound 2.

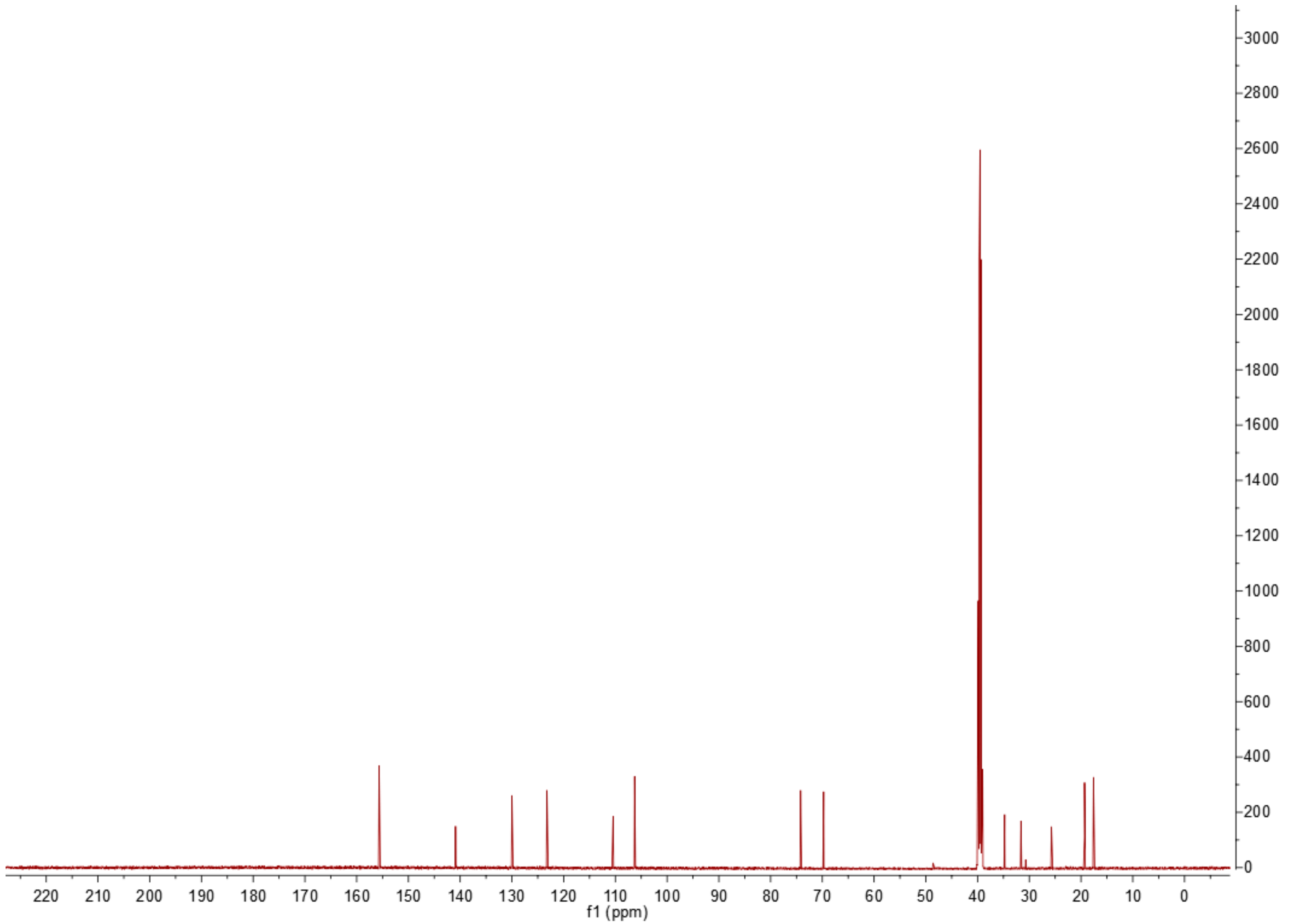

Figure S7: ^{13}C NMR spectrum (125 MHz, DMSO-d) of compound 2.

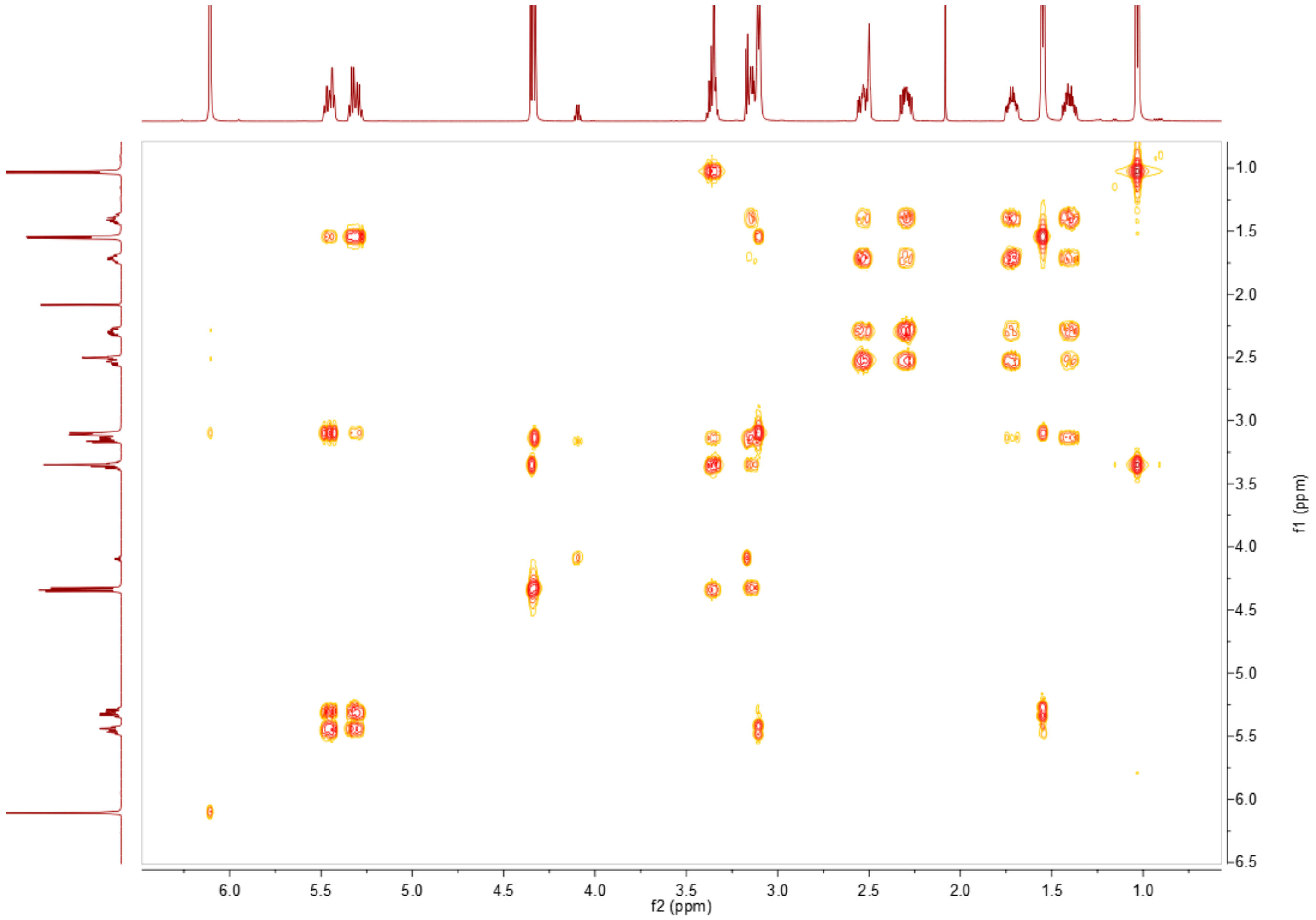

Figure S8: COSY NMR spectrum (500 MHz, DMSO -d) of compound 2.

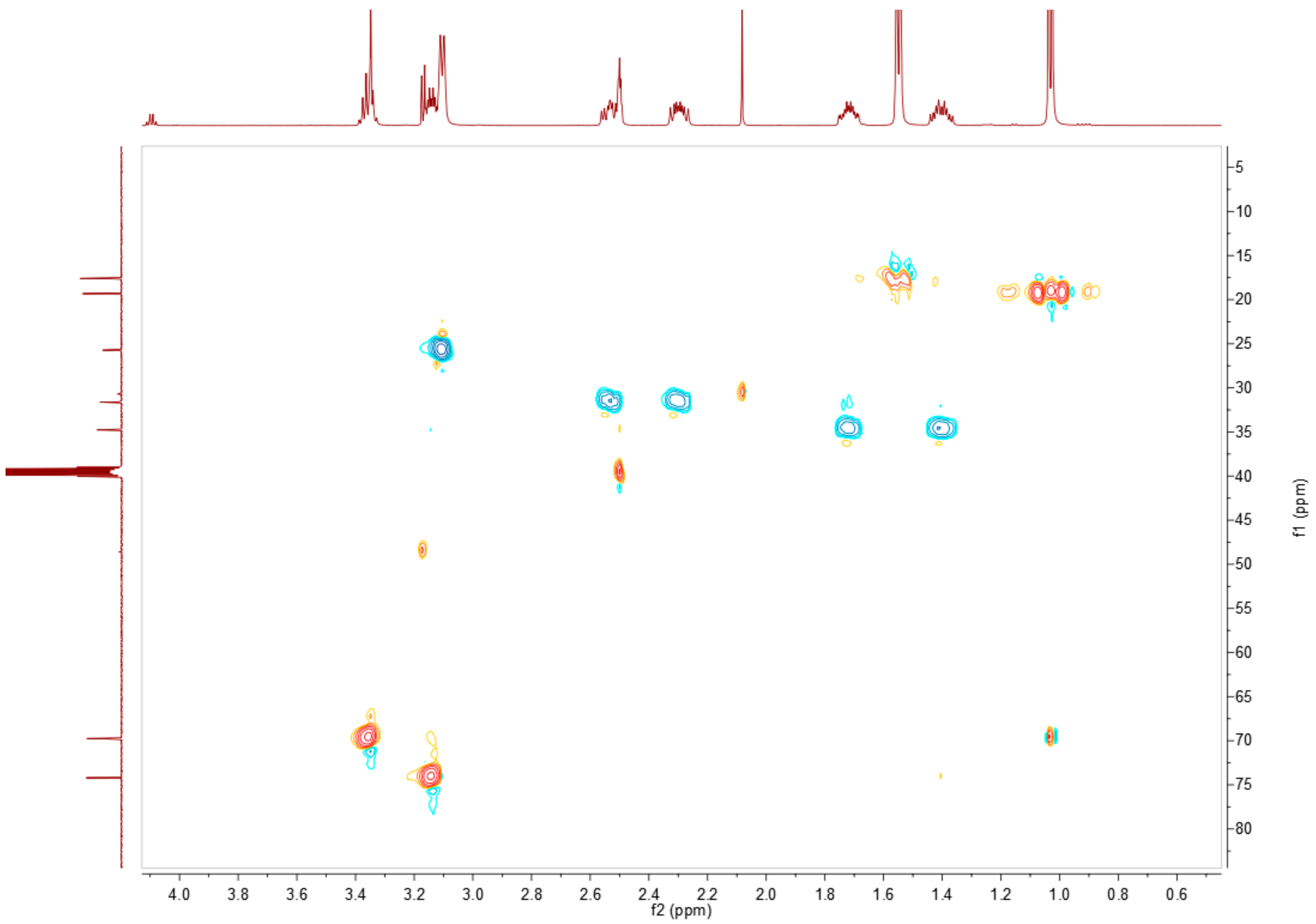

Figure S9: HSQC NMR spectrum (500 MHz, DMSO-d) of compound 2.

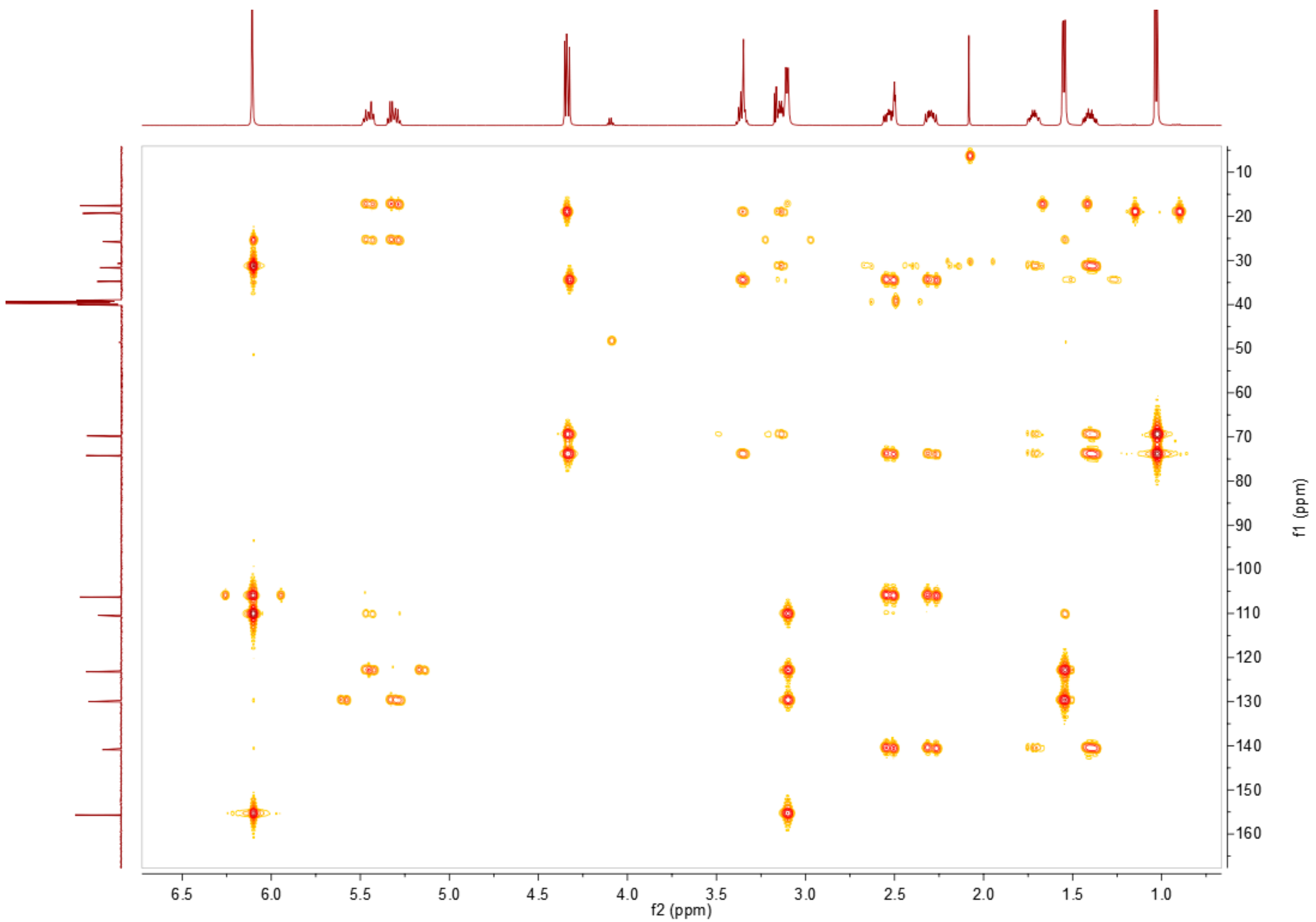

Figure S10: HMBC NMR spectrum (500 MHz, DMSO-d) of compound 2.

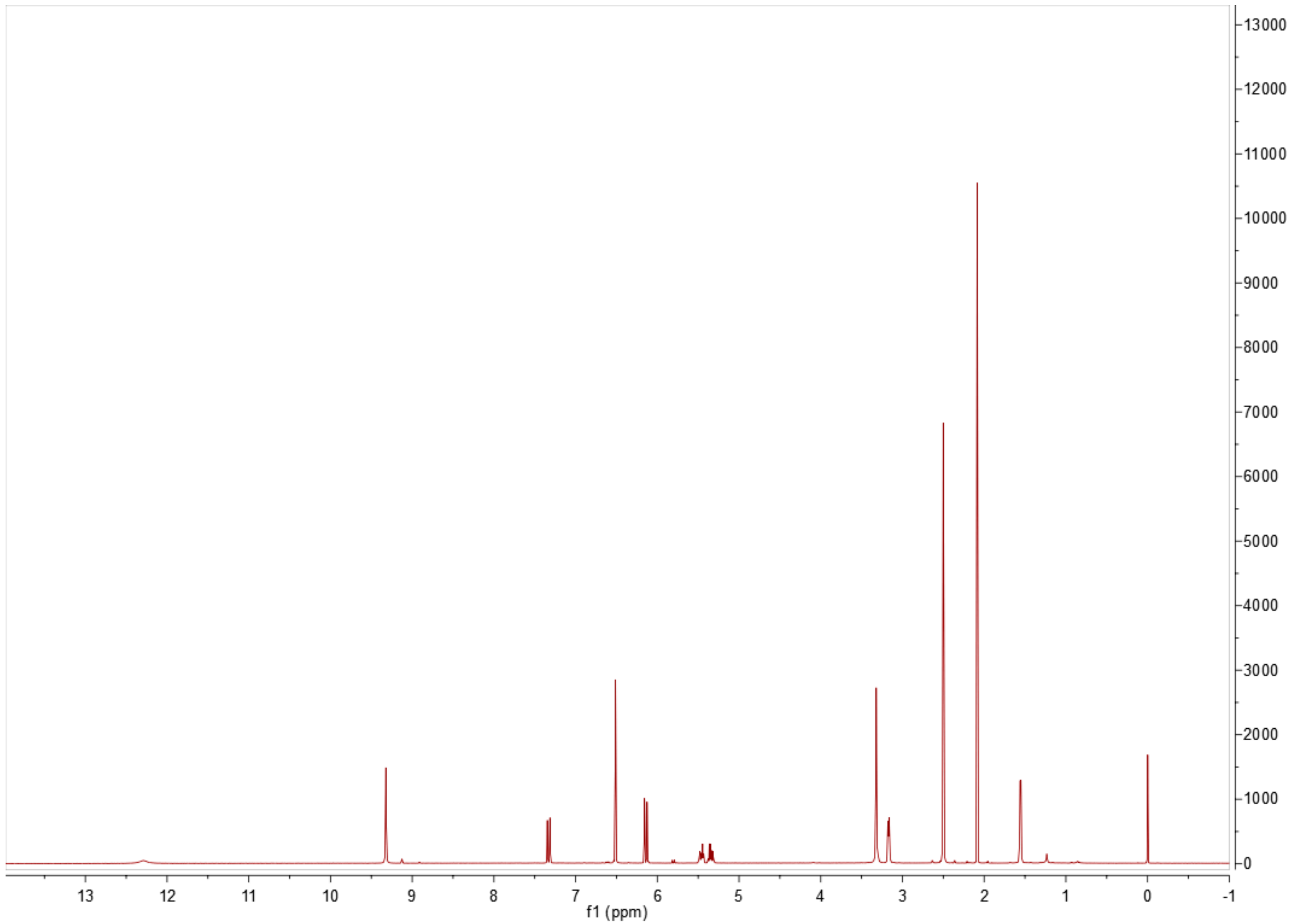

Figure S11: ^{1}H NMR spectrum (500 MHz, DMSO-d) of compound 3.

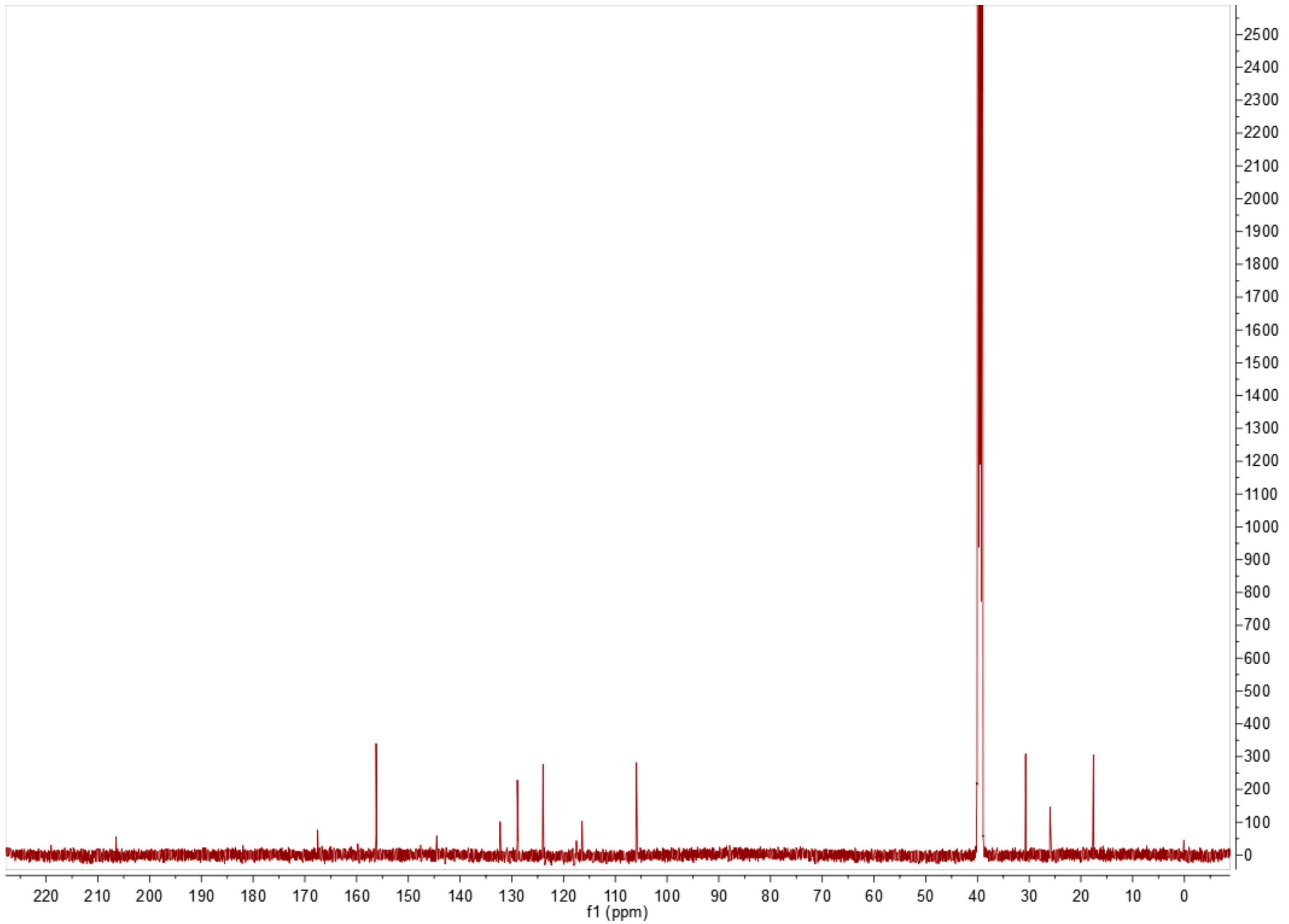

Figure S12: ^{13}C NMR spectrum (125 MHz, DMSO-d) of compound 3.

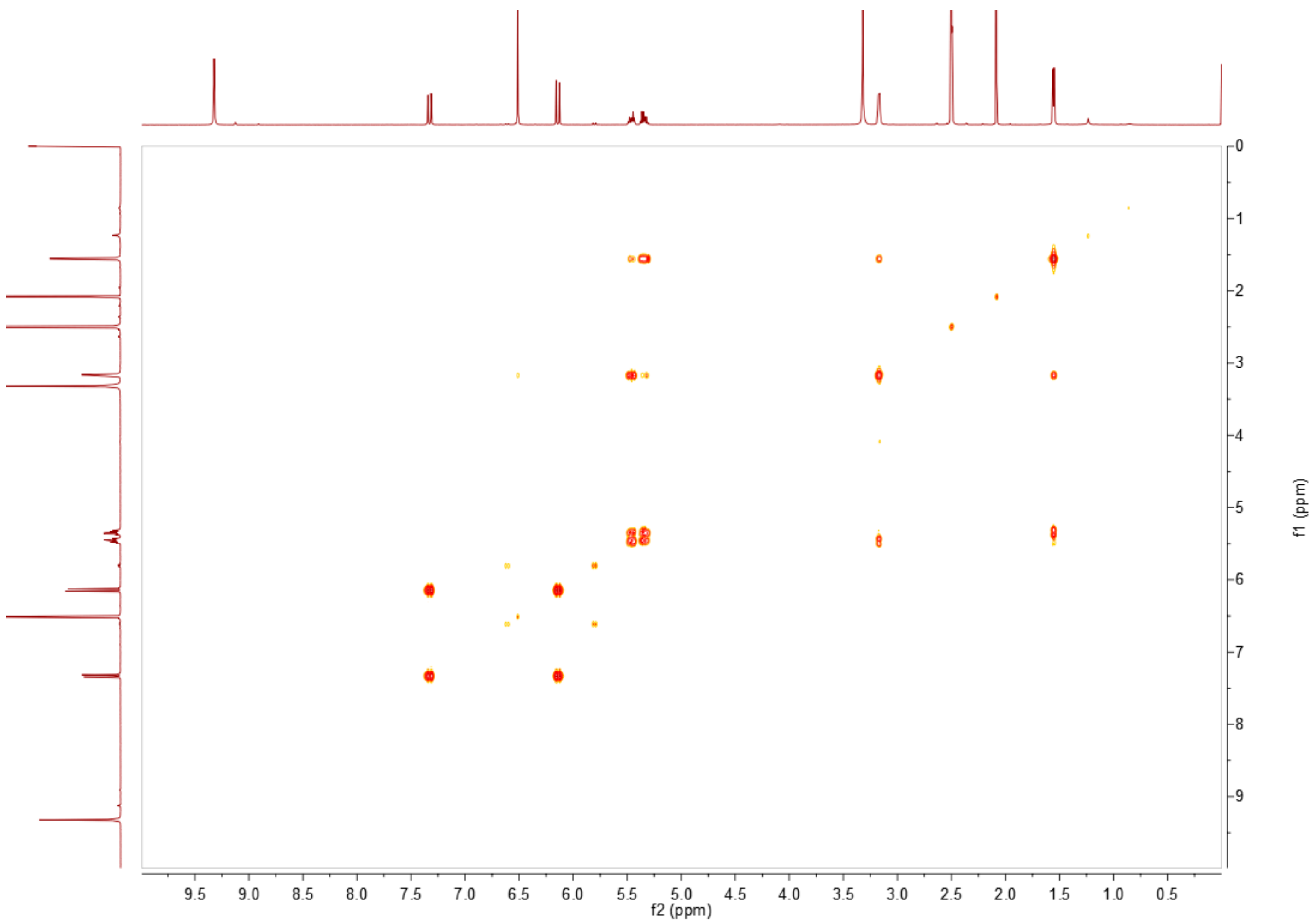

Figure S13: COSY NMR spectrum (500 MHz, DMSO -d) of compound 3.

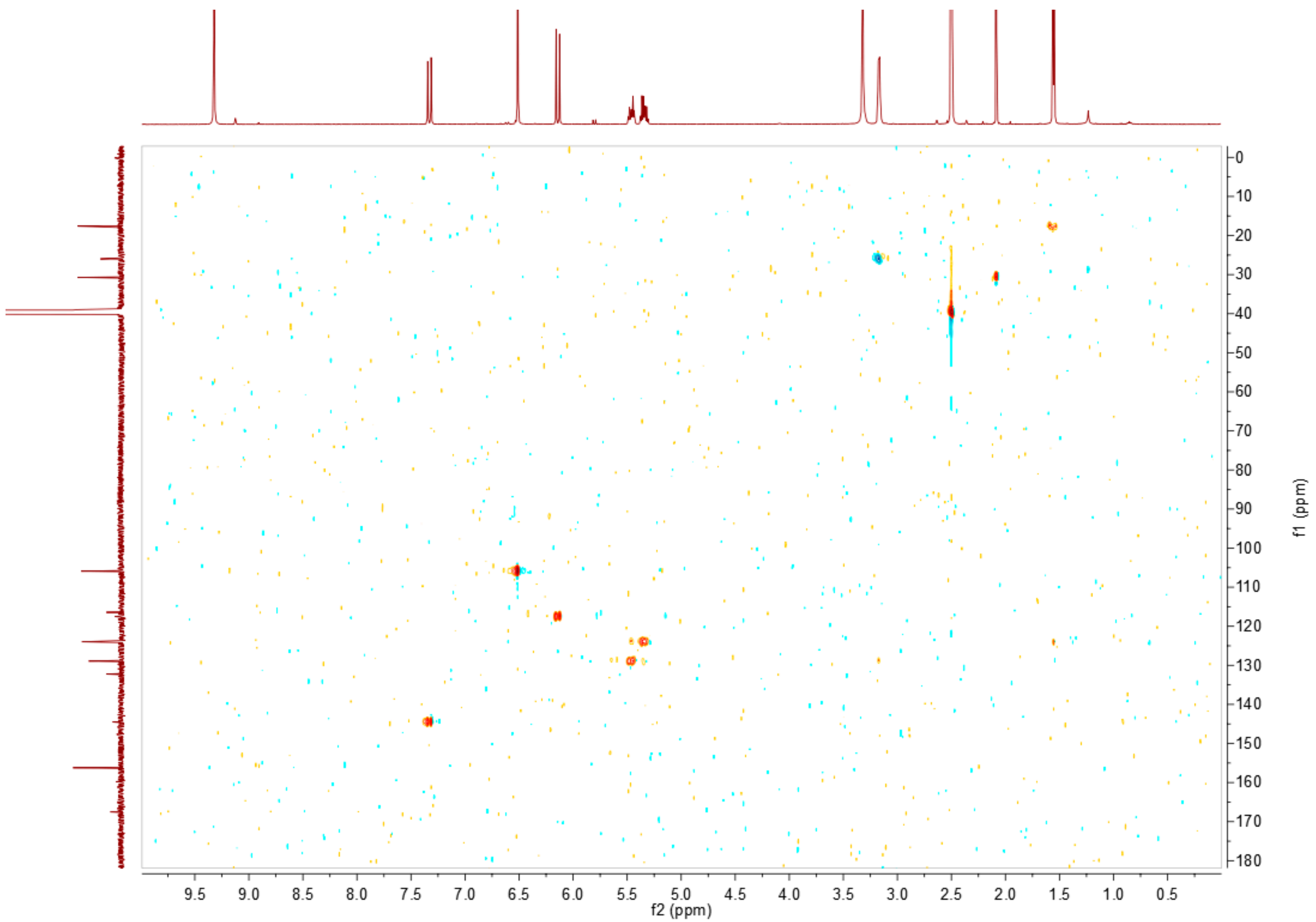

Figure S14: HSQC NMR spectrum (500 MHz, DMSO-d) of compound 3.

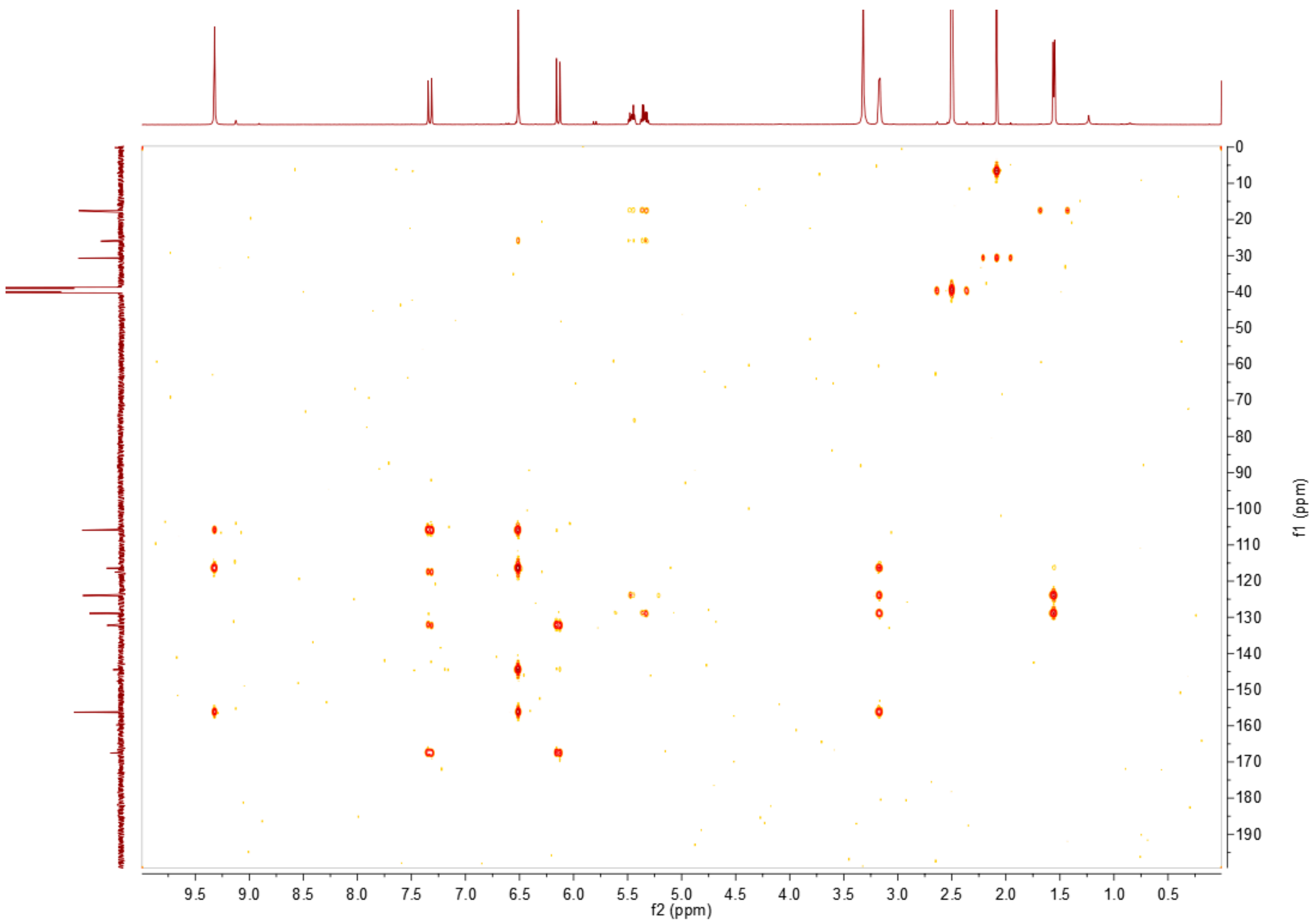

Figure S15: HMBC NMR spectrum (500 MHz, DMSO-d) of compound 3.

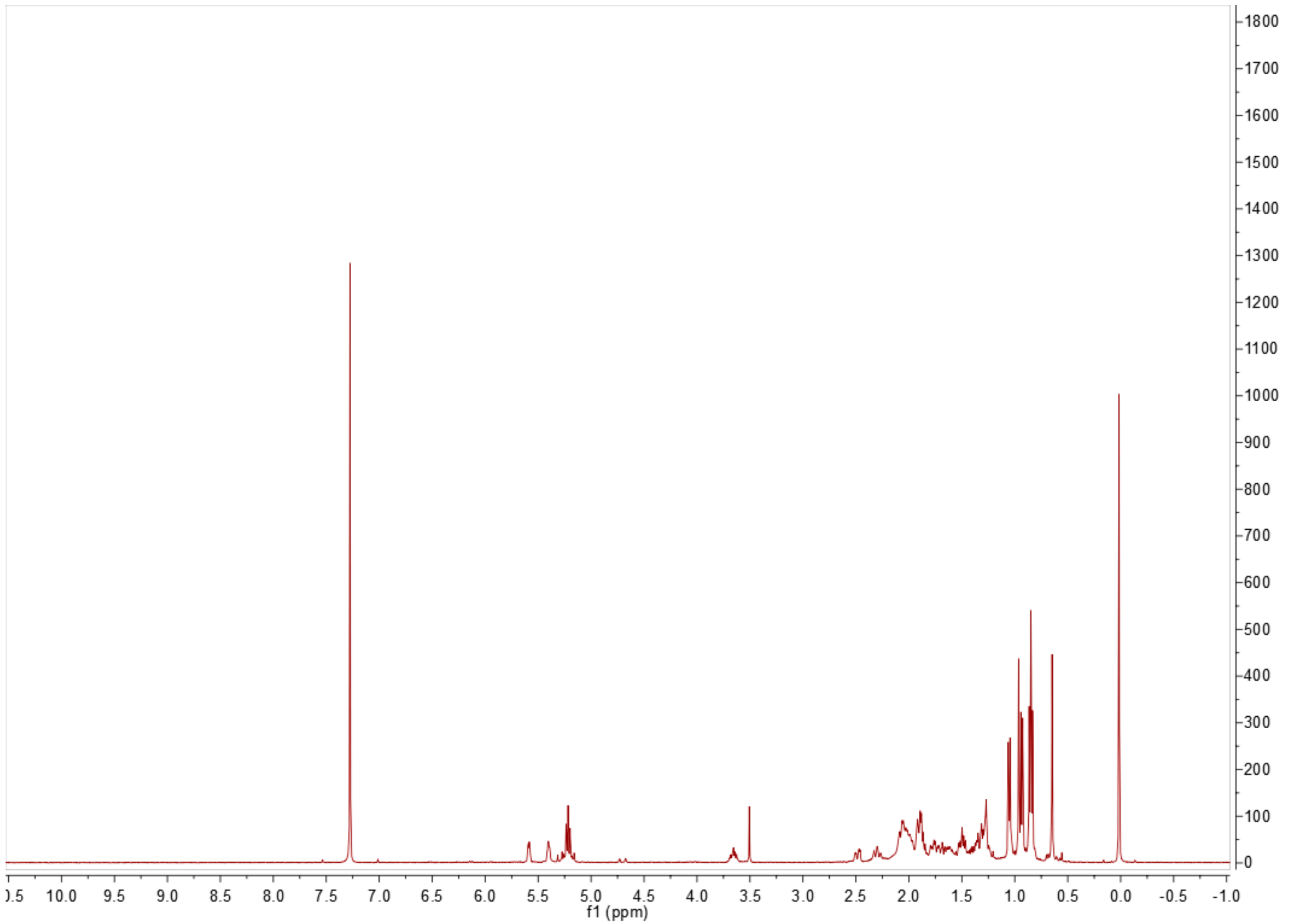

Figure S16: ^{1}H NMR spectrum (400 MHz, Chloroform-d) of compound 4.

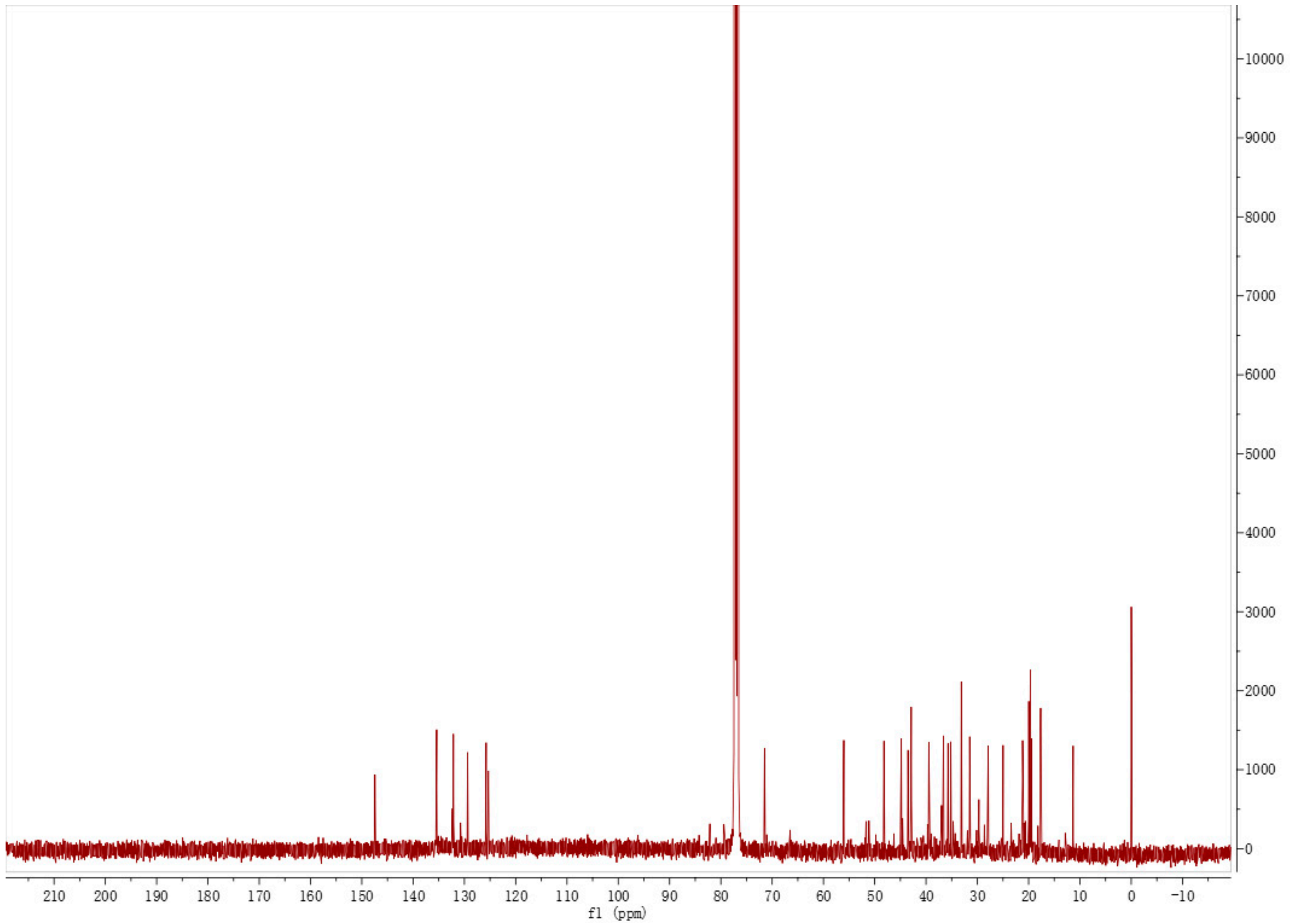

Figure S17: ^{13}C NMR spectrum (100 MHz, Chloroform-d) of compound 4.

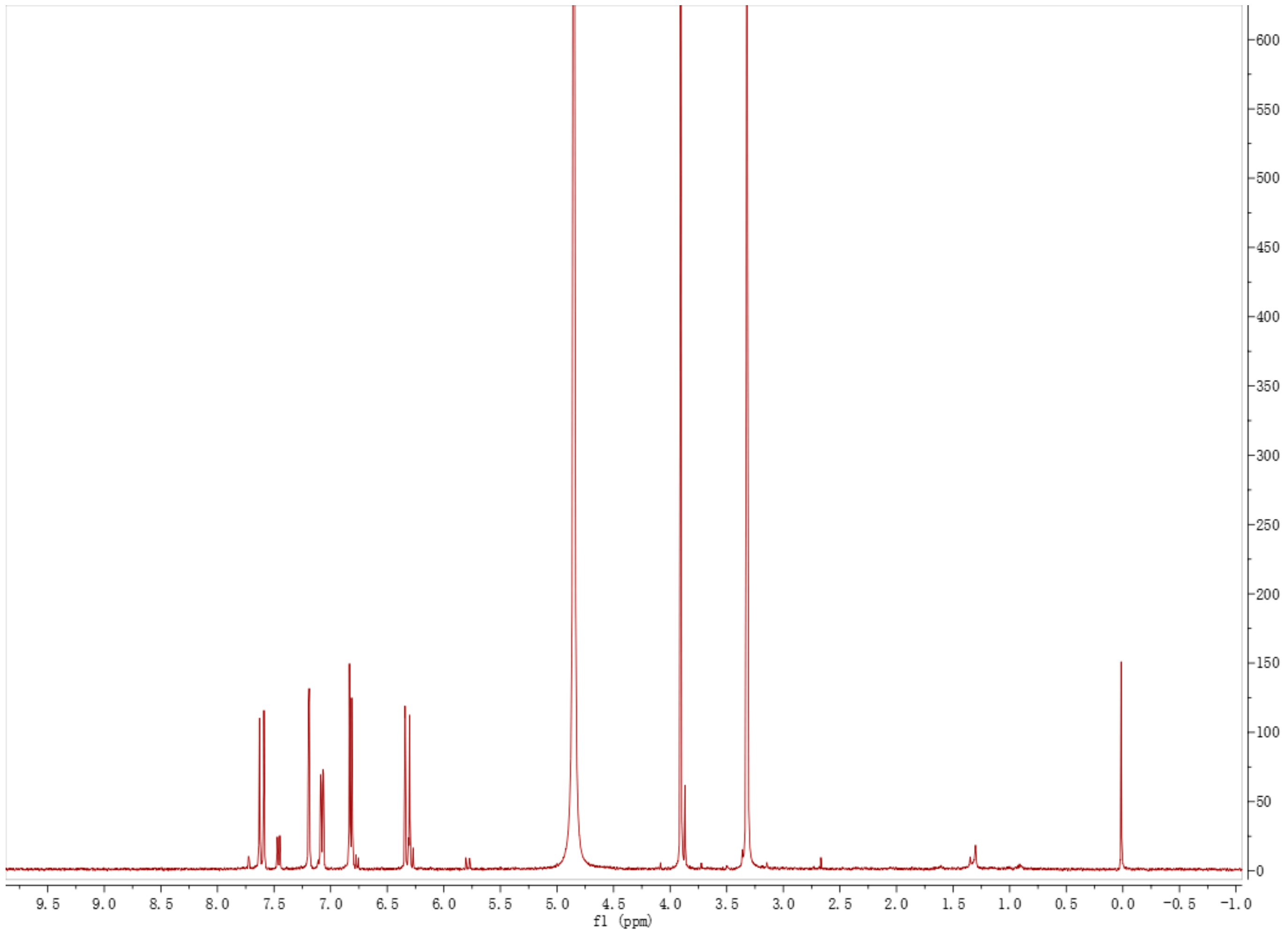

Figure S18: ^{1}H NMR spectrum (400 MHz, Methanol-D4) of compound 5.

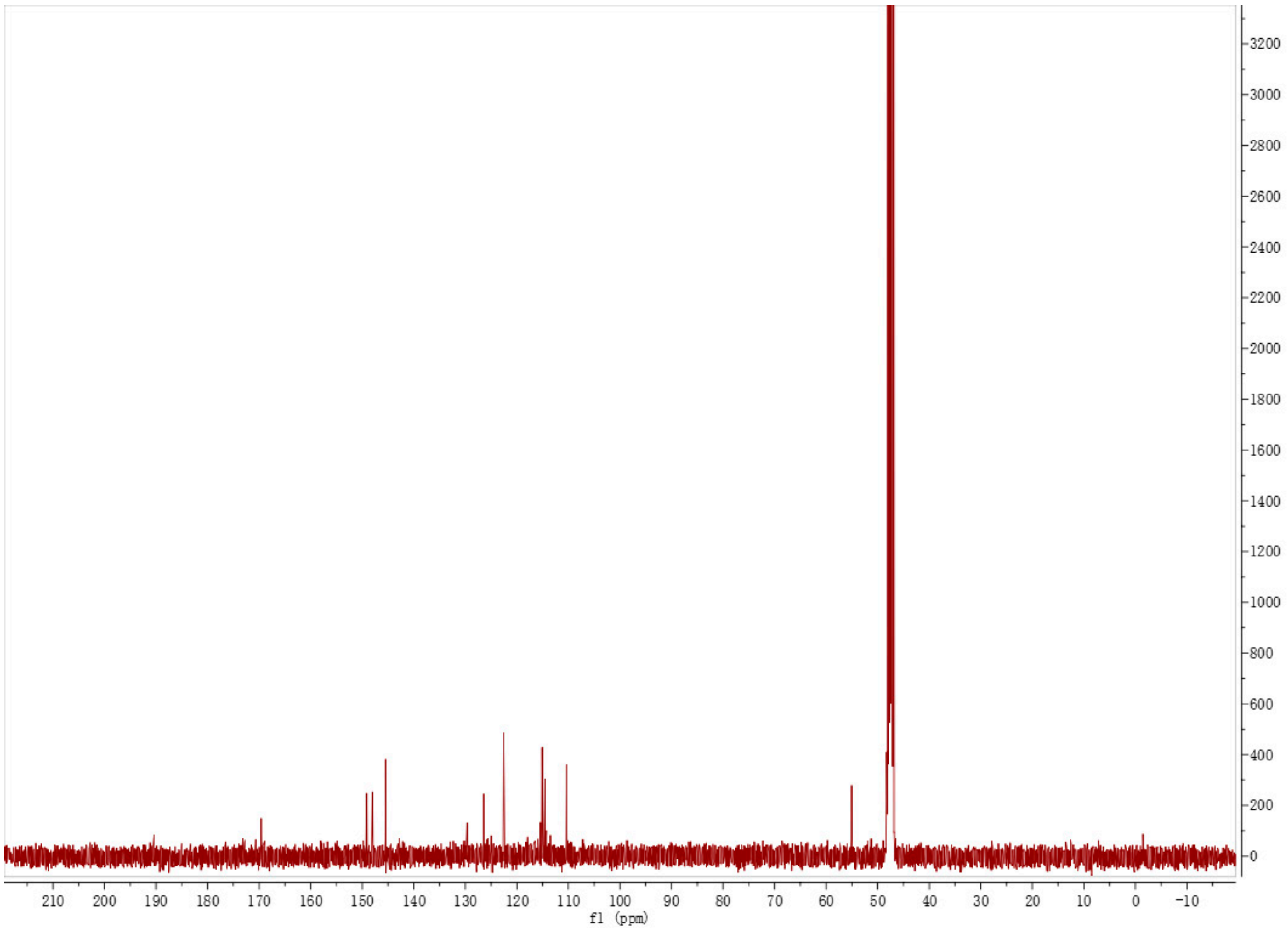

Figure S19: ^{13}C NMR spectrum (100 MHz, Methanol-D4) of compound 5.

Table S5: Accession numbers of sequences used in the phylogenetic analyses.

Taxa	Strain	GenBank Accession Numbers			
		ITS	LSU	SSU	TEF
Chaetosphaeronema achilleae	MFLUCC 16-0476	NR_153927	KX765266		
Chaetosphaeronema hispidulum	CBS 216.75	KF251148	EU754144	EU754045	
Dematiopleospora cirsii	MFLUCC 15-0615	KX274243	KX274250		KX284708
Dematiopleospora fusiformis	MFLU 15-2133	NR_157463	NG_069422	NG_065662	
Dermatiopleospora mariae	MFLUCC 13-0612	KX274244	KJ749653	KJ749652	KJ749655
Dlhawksworthia alliariae	MFLUCC 13-0070	KX494876	KX494877	KX494878	
Neostagonospora artemisiae	MFLUCC 17-0693	MG828929	MG829038	NG_063688	
Nodulosphaeria guttulatum	MFLUCC 15-0069	KY496746	KY496726	KY501115	KY514394
Nodulosphaeria multiseptata	MFLUCC 15-0078/IT1889	KY496748	KY496728	KY501116	KY514396
Nodulosphaeria scabiosae	MFLUCC 14-1111	KU708850	KU708846	KU708842	KU708854
Ophiobolus artemisiae	MFLUCC 14-1156	KT315508	KT315509	MG520979	MG520905
Ophiobolus artemisiae	MFLU 15-1966	MG520940	MG520960	MG520978	MG520904
Ophiobolus artemisiicola	MFLU 15-2137	NR_157527	NG_059871	NG_065147	MG829220
Ophiobolus artemisiicola	MFLU 15-2140	MG828931	MG829040	MG829146	MG829221
Ophiobolus disseminans	MFLUCC 17-1787	MG520941	MG520961	MG520980	MG520906

Ophiobolus disseminans	MFLUCC 17-1790	MG520942	MG520962	MG520981	MG520907
Ophiobolus ponticus	MFLUCC 17-2273	MG520943	MG520963	MG520982	MG520908
Ophiobolus rossicus	MFLU 17-1639	MG520944	MG520964	MG520983	MG520909
Ophiobolus rudis	CBS 650.86	KY090650	GU301812	AF164356	GU349012
Ophiobolus senecionis	MFLUCC 13-0575	KT728365	KT728366		
Ophiobolus malleolus	MFLUCC 15-1077	MK356377	MK356351	MK356365	
Paraophiobolus arundinis	MFLUCC 17-1789	MG520945	MG520965	MG520984	MG520912
Paraophiobolus plantaginis	MFLUCC 17-0245	KY797641	KY815010	KY815012	MG520913
Pseudoophiobolus achilleae	MFLU 17-0925	MG520946	MG520966		
Pseudoophiobolus galii	MFLUCC 17-2257	MG520947	MG520967	MG520989	MG520926
Pseudoophiobolus italicus	MFLUCC 17-2255	MG520948	MG520968	MG520990	MG520927
Pseudoophiobolus mathieui	MFLUCC 17-1785	MG520951	MG520971	MG520992	MG520929
Paraphoma radicina	CBS 111.79	KF251172	KF251676	EU754092	GU349076
Ophiobolus hydei	YG-S81-1	MK981300	MK981300	MK981300	MK981300
Ophiobolus hydei	YG-S82-2	MK981301	MK981305	MK981303	
Macroventuria anomochaeta	CBS 525.71	GU237881	GU456315	GU238208	GU456262
